# The potent and selective cyclin-dependent kinases 4 and 6 inhibitor ribociclib (LEE011) is a versatile combination partner in preclinical cancer models

**DOI:** 10.18632/oncotarget.26215

**Published:** 2018-10-16

**Authors:** Sunkyu Kim, Ralph Tiedt, Alice Loo, Thomas Horn, Scott Delach, Steven Kovats, Kristy Haas, Barbara Schacher Engstler, Alexander Cao, Maria Pinzon-Ortiz, Iain Mulford, Michael G. Acker, Rajiv Chopra, Christopher Brain, Emmanuelle di Tomaso, William R. Sellers, Giordano Caponigro

**Affiliations:** ^1^ Novartis Institutes for BioMedical Research, Oncology Disease Area, Cambridge, MA, USA; ^2^ Novartis Institutes for BioMedical Research, Oncology Disease Area, Basel, Switzerland, USA; ^3^ Novartis Institutes for BioMedical Research, Chemical Biology & Therapeutics, Cambridge, MA, USA; ^4^ Novartis Institutes for BioMedical Research, Global Discovery Chemistry, Cambridge, MA, USA

**Keywords:** CDK4/6 inhibitor, ribociclib, LEE011, preclinical, selectivity

## Abstract

Inhibition of cyclin-dependent kinases 4 and 6 (CDK4/6) is associated with robust antitumor activity. Ribociclib (LEE011) is an orally bioavailable CDK4/6 inhibitor that is approved for the treatment of hormone receptor–positive, human epidermal growth factor receptor 2–negative advanced breast cancer, in combination with an aromatase inhibitor, and is currently being evaluated in several additional trials. Here, we report the preclinical profile of ribociclib.

When tested across a large panel of kinase active site binding assays, ribociclib and palbociclib were highly selective for CDK4, while abemaciclib showed affinity to several other kinases. Both ribociclib and abemaciclib showed slightly higher potency in *CDK4*-dependent cells than in *CDK6*-dependent cells, while palbociclib did not show such a difference. Profiling CDK4/6 inhibitors in large-scale cancer cell line screens *in vitro* confirmed that *RB1* loss of function is a negative predictor of sensitivity. We also found that routinely used cellular viability assays measuring adenosine triphosphate levels as a proxy for cell numbers underestimated the effects of CDK4/6 inhibition, which contrasts with assays that assess cell number more directly. Robust antitumor efficacy and combination benefit was detected when ribociclib was added to encorafenib, nazartinib, or endocrine therapies in patient-derived xenografts.

## INTRODUCTION

A hallmark of cancer is unchecked cell division. Dysregulation of cell-cycle control in cancer often occurs through disruption of cell-cycle checkpoint regulators [1–4]. The product of *RB1* is one such protein guarding the entry into S phase. In its quiescent (nonphosphorylated) state, retinoblastoma protein (Rb) binds to the E2 transcription factor (E2F) family members creating a transcriptional repression complex that is sufficient to arrest cells in G_1_ [2, 3, 5–7]. Activation of mitogenic signaling pathways via exogenous stimuli and/or genetic lesions, such as the RAS/RAF/MEK/ERK (MAPK), PI3K, and hormone receptor (HR) pathways, results in increased expression of D cyclins [6]. The D cyclins then bind to and activate cyclin-dependent kinases 4 and 6 (CDK4/6), which in turn phosphorylate Rb and leads to the release of E2F proteins [2, 3, 6] and derepression of E2F-dependent promotors [7]. Released E2F proteins activate genes required for S phase entry and DNA replication [6].

Acquired genetic aberrations that increase CDK4/6 activity include (1) deletion or silencing of *CDKN2A*, which encodes p16, a cellular inhibitor of CDK4/6, (2) amplification and translocation of D cyclin genes, (3) CDK4/6-activating mutations that block the binding of p16, and (4) amplification of *CDK4* and *CDK6* [4, 8, 9]. Equally important are genetic aberrations in signaling pathways upstream of D cyclins, such as *RAS* mutations (*KRAS* and *NRAS*), the *BRAF* V600E mutation, *MEK* mutation, *PI3KCA* mutation, and *PTEN* deletion, which then result in elevated levels of D cyclins with subsequent CDK4/6 activation [4, 5, 8].

Many studies have since demonstrated the requirement of CDK4/6 in numerous solid tumors and hematologic malignancies, particularly breast cancer [10–17]. When mice harboring mammary tumors overexpressing the *ERBB2* gene were crossed with CDK4 knockout mice, the resulting littermates did not develop tumors, and the acute loss of cyclin D1 or CDK4 proteins mediated via RNA interference attenuated tumor growth. These data suggest that cyclin D1 and CDK4 are required in some contexts for tumor initiation and maintenance [17]. In addition, when a panel of 47 human breast cancer and immortalized breast cell lines grown *in vitro* were treated with a CDK4/6 inhibitor (palbociclib), sensitive cell lines (estrogen receptor positive) showed increased expression of the genes *RB1* and *CCND1* and reduced expression of *CDKN2A* (p16) [11]. In the context of these results, several small-molecule inhibitors are currently approved or under development to treat HR-positive, advanced-stage breast cancer [2, 3, 10].

Ribociclib (LEE011) is a highly selective, orally bioavailable CDK4/6 inhibitor that has recently been approved for the treatment of HR-positive, human epidermal growth factor receptor 2 (HER2)–negative advanced breast cancer in combination with an aromatase inhibitor [18, 19]. In the Phase 3 MONALEESA-2 trial, ribociclib in combination with letrozole as first-line treatment significantly improved progression-free survival, with a manageable safety profile, compared with letrozole alone [20]. To further characterize ribociclib, we report its selectivity in biochemical assays, cellular activity, and *in vivo* activity in both single-agent and combination settings.

## RESULTS

### Ribociclib is a potent, selective inhibitor of CDK4/6

**Figure 1:**
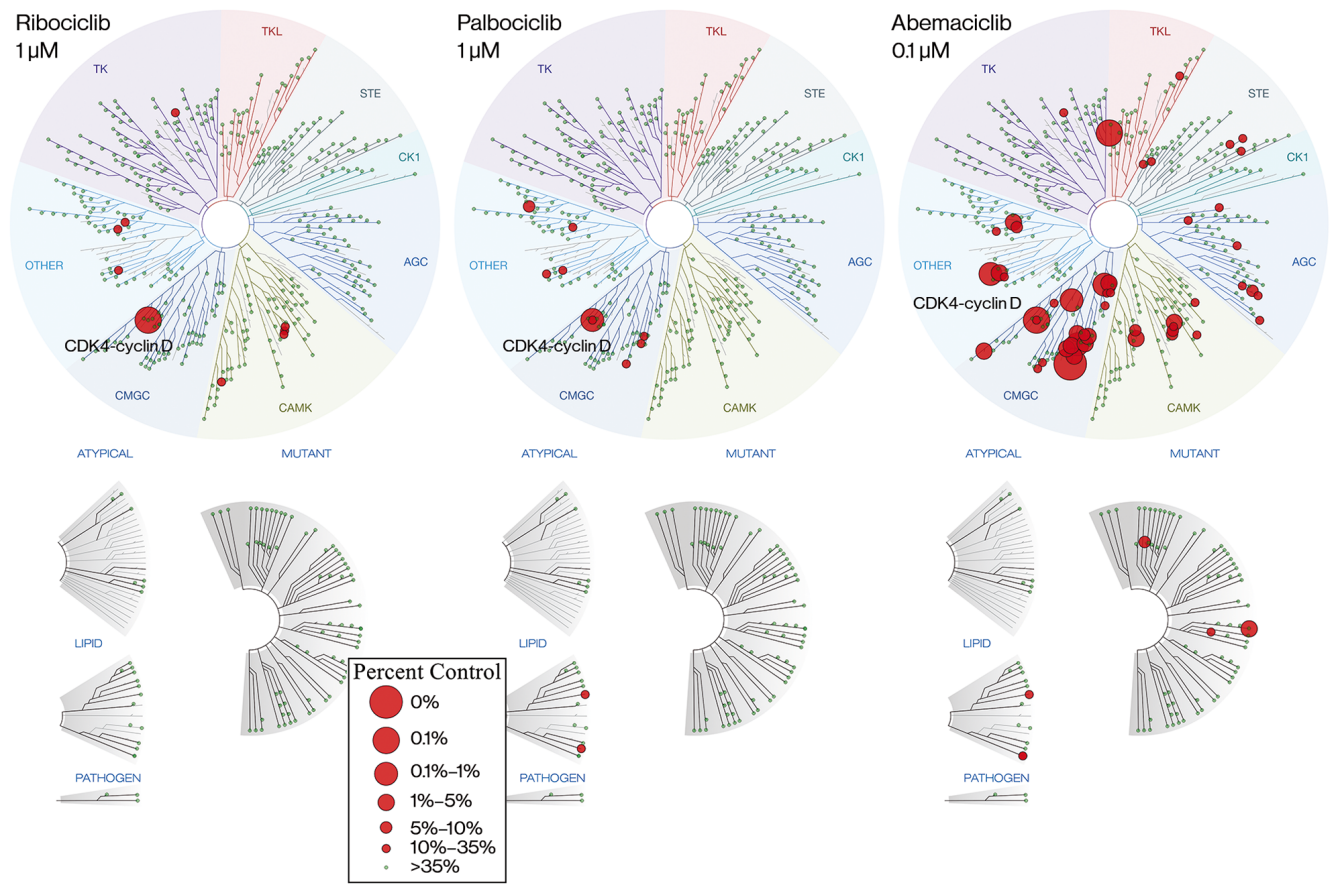
Ribociclib is a highly selective CDK4/6 inhibitor TREE*spot* view of KINOME*scan*^©^ selectivity panel for ribociclib and palbociclib at 1 μM and abemaciclib at 0.1 μM. Abemaciclib is shown at a 10-fold lower concentration than ribociclib and palbociclib because of its greater affinity for CDK4 (CDK6 was not part of the selectivity panel). Kinases that bind are marked with red circles if <35% of the respective recombinant kinase remained captured on the immobilized ligand in the presence of the indicated concentration of CDK4/6 inhibitor, relative to a DMSO control. Larger circles indicate a higher affinity of binding. AGC, cAMP-dependent, cGMP-dependent, and protein kinase C; CDK, cyclin-dependent kinase; CK, creatine kinase; CMGC, cyclin-dependent, mitogen-activated glycogen synthase and CDK-like kinase; DMSO, dimethyl sulfoxide; STE, yeast sterile kinase; TK, thymidine kinase; TKL, tyrosine kinase-like.

Ribociclib (7-cyclopentyl-2-(5-piperazin-1-yl-pyridin-2-ylamino)-7H-pyrrolo[2,3-d]pyrimidine-6-carboxylic acid dimethylamide) inhibits the enzymatic activity of CDK4-cyclin D1 and CDK6-cyclin D3 complexes with half-maximal inhibitory concentrations (IC_50_) of 0.01 and 0.039 μM, respectively, and is far less active against CDK1/cyclin B, CDK2/cyclin A, CDK5/p25, and CDK9/cyclin T1 complexes [21, 22]. To further characterize the selectivity of ribociclib and compare it to the selectivity of the CDK4/6 inhibitors palbociclib and abemaciclib, the affinities of these molecules for CDK4 ± cyclin D1/D3 and 465 other kinases and disease-relevant mutant variants were analyzed using the KINOME*scan*^*®*^ selectivity screening platform (Supplementary Table 1A-1C). This analysis indicated that ribociclib and palbociclib have similar affinities for CDK4, which were ∼10-fold lower than that observed for abemaciclib (Supplementary Table 1A). However, it is interesting to note that all 3 molecules had a substantially reduced affinity for unbound CDK4 compared with CDK4-cyclin D. The 3 CDK4/6 inhibitors were screened in the full platform at 0.1 and 1.0 μM (Figure 1, Supplementary Table 1B-1C, and Supplementary Figure 1). When ribociclib was tested in the kinase selectivity screen at 0.1 μM, only CDK4-cyclin D1 and CDK4-cyclin D3 showed a reduction by >65%, a commonly used cutoff to define hits in this screen (CDK6 was not part of the selectivity panel). At 1.0 μM, only 8 additional hits were detected (Figure 1). Palbociclib displayed a similarly low hit rate, with 2 and 9 kinases other than CDK4 identified to be bound at 0.1 μM and 1.0 μM, respectively. In line with previous reports, and in contrast with ribociclib and palbociclib, abemaciclib was less selective [23, 24], producing 53 and 115 kinase binding signals in addition to CDK4 at 0.1 μM and 1.0 μM, respectively (Figure 1 and Supplementary Figure 1). In line with the reduced affinity for CDK4 in the absence of cyclin D, unbound CDK4 did not reach the cutoff of >65% binding reduction with ribociclib (48% reduction at 1 μM), while it exceeded the cutoff with palbociclib and abemaciclib (69% reduction at 1 μM and 83% reduction at 100 nM, respectively; Supplementary Table 1B). In contrast, all 3 molecules dissociated cyclin D–bound CDK4 from the capture matrix by >99% at these concentrations. Collectively, these data show that ribociclib and palbociclib are highly selective for CDK4 relative to other kinases, whereas abemaciclib is more promiscuous, even when accounting for differences in affinity for CDK4.

### Ribociclib is more active in *CDK4*-dependent than *CDK6*-dependent cell lines

The CDK4/6 inhibitor abemaciclib has been reported to exhibit slightly better selectivity for CDK4 over CDK6 [24, 25]. Interestingly, we saw a similar differential of CDK4 versus CDK6 activity in our biochemical assays with ribociclib. To assess this differential in a cellular context, the activities of ribociclib, palbociclib, and abemaciclib were tested in proliferation assays using cancer cell lines where either CDK4 or CDK6 plays a dominant role for cell-cycle progression. To identify such cell lines, we used 2 data sets: a large-scale pooled short hairpin RNA (shRNA) screen across 398 cell lines interrogating cell-autonomous dependencies of 7837 genes targeted by 20 shRNAs each [26], and mRNA expression levels as determined by Affymetrix™ U133 Plus 2.0 arrays for the Cancer Cell Line Encyclopedia [27] (Supplementary Table 2). Cell lines in which *CDK4* knockdown but not *CDK6* knockdown had a pronounced effect on growth were enriched for those derived from breast cancers. In addition, most *CDK4*-dependent lines were also *CCND1* dependent and had strikingly low mRNA expression of *CDK6* (Supplementary Figure 2A-2C). In contrast, *CDK6* dependence was almost exclusively seen in cancer cell lines of hematopoietic or lymphoid origin, and these lines also often showed *CCND3* dependence. Unlike the *CDK4*-dependent cell lines that had little or no *CDK6* mRNA expression, *CDK6*-dependent lines typically expressed *CDK4* mRNA, although several of these lines showed very low *CCND1* mRNA expression (Supplementary Figure 2C and 2D). On the basis of these observations, we selected 4 cell lines in which *CDK4* was likely dominant over *CDK6* (JeKo-1, a mantle cell lymphoma line with cyclin D1 translocation, and CAMA-1, MCF-7 and T47D, which are HR-positive breast cancer cell lines) and 4 *CDK6-*dependent cell lines of hematopoietic or lymphoid origin (SEM, REH, MOLM-13, and Pfeiffer). We then determined IC_50_ values for ribociclib, palbociclib, and abemaciclib using the CyQuant cell proliferation assay (ThermoFisher Scientific). As shown in Table 1, values for palbociclib were comparable in all 8 cell lines. Ribociclib showed greater activity in *CDK4*-dependent than *CDK6*-dependent cell lines, and this difference was greater than that seen in biochemical assays. In line with previous reports [25], abemaciclib was also more active in *CDK4*-dependent cell lines.

**Table 1:** IC_50_ Values of CDK4/6 Inhibitors

Cell line	Cancer type	Dominant CDK	Ribociclib IC_50_, mean ± SD, μM	Palbociclib IC_50_, mean ± SD, μM	Abemaciclib IC_50_, mean ± SD, μM
JeKo-1	MCL	CDK4	143 ± 87	72 ± 33	20 ± 9
CAMA-1	ER+ BC	CDK4	162 ± 59	50 ± 24	28 ± 2
MCF-7	ER+ BC	CDK4	62 ± 30	30 ± 18	11 ± 7
T47D	ER+ BC	CDK4	111 ± 14	66 ± 19	13 ± 3
REH	ALL	CDK6	1030 ± 246	60 ± 17	72 ± 6
SEM	ALL	CDK6	1484 ± 215	87 ± 28	162 ± 37
Pfeiffer	DLBCL	CDK6	948 ± 53	89 ± 32	66 ± 25
MOLM-13	AML	CDK6	365 ± 62	47 ± 25	57 ± 21

IC_50_values (mean ± SD) of ribociclib, palbociclib, and abemaciclib were determined using the CyQuant cell proliferation assay. The average differential for CDK4 versus CDK6 dependent lines for ribociclib, palbociclib, and abemaciclib is 8.0-, 1.3-, and 5.5-fold, respectively. Abbreviations: ALL, acute lymphoblastic leukemia; AML, acute myeloid leukemia; BC, breast cancer; CDK, cyclin-dependent kinase; DLBCL, diffuse large B-cell lymphoma; ER+, estrogen receptor–positive; IC_50_, half-maximal inhibitory concentration; MCL, mantle-cell lymphoma; SD, standard deviation.

### *In vitro* screening in a large panel of cell lines identifies *RB1* as a major determinant of sensitivity to ribociclib

Ribociclib induces dephosphorylation of phosphorylated Rb (pRb) and concomitant G_1_ cell-cycle arrest in *RB1*-proficient JeKo-1 mantle cell lymphoma cells, while no effect on cell-cycle progression was observed in the *RB1*-deleted lung adenocarcinoma cell line NCI-H2009 up to 10 μM (Brain C et al, manuscript in preparation). To determine whether this observation extended to other cancer cell types, we profiled ribociclib and palbociclib across a panel of nearly 500 cancer cell lines of mixed lineage within the context of the Cancer Cell Line Encyclopedia project [27]. In this standardized high-throughput experiment, cells were exposed to compounds for 3 days, and cell number was quantified by measuring cellular ATP levels using CellTiter-Glo^®^ (CTG; Promega). Surprisingly, the effect of both CDK4/6 inhibitors was modest on average across cell lines (Supplementary Table 3), which in some instances contrasted with previous data obtained in other assay formats. Nonetheless, several interesting observations were made. First, consistent with prior reports [28], neuroblastoma and malignant rhabdoid tumor–derived cell lines were among the most sensitive cell lines examined [29]. *in vivo* efficacy studies further confirmed the activity of ribociclib in such cancer models (Supplementary Figure 3A and 3B). Second, when stratifying cell lines by the status of *RB1*, we observed a significant difference, with *RB1*-proficient cells being more sensitive to both CDK4/6 inhibitors (Supplementary Figure 3C). The distinction was most pronounced when considering the maximal effect level (A_max_) as a measure of sensitivity. This observation is in line with results from a similar large cell line screen [30] and supports the hypothesis that functional Rb is necessary to mediate the antiproliferative effects of CDK4/6 inhibitors. In our screen, a positive correlation between A_max_ of ribociclib and palbociclib (Supplementary Figure 3D) suggest that both CDK4/6 inhibitors have similar activity *in vitro*.

### *In vitro* antiproliferative effects of CDK4/6 inhibition reveal efficacy across many different lineages that can be masked by morphological changes

To understand why some cell line sensitivity data obtained in high throughput CTG format, which uses ATP levels as a proxy for cell numbers, disagreed with previous proliferation data obtained via other methods such as cell counting, bromodeoxyuridine incorporation, or flow cytometry assays (data not shown), we evaluated the effects of ribociclib more closely in 3 neuroblastoma lines where CTG measurements agreed with alternate assay formats and 3 melanoma cell lines where CTG measurements did not.

**Figure 2:**
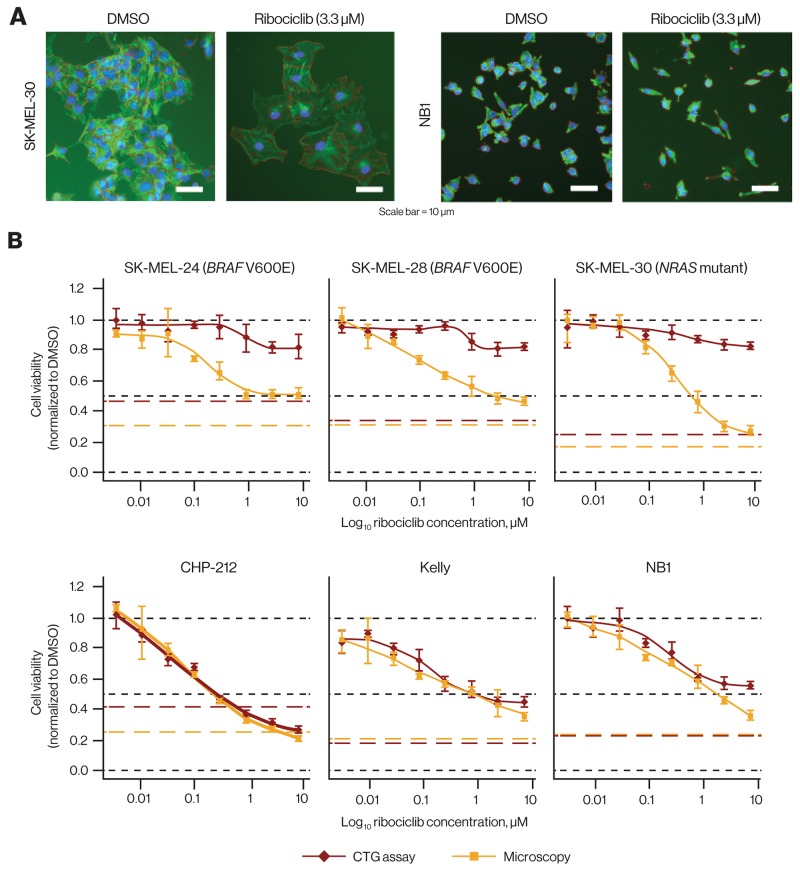
Discrepancy of ribociclib effects *in vitro* comparing ATP quantification and microscopy **(A)** Cells after 72-hour ribociclib treatment (3.3 μM) vs DMSO. Ribociclib treatment affected the number of NB1 cells without apparent size changes and resulted in increased size of SK-MEL-30 cells accompanied by a reduction in number (original magnification × 10; tubulin [green] and DNA/nuclei [blue] stained with anti–-α-tubulin FITC antibody and Hoechst 33342, respectively). **(B)** Dose-response curves comparing cell viability by ATP quantification and microscopy in 3 melanoma cell lines (upper panel) and 3 neuroblastoma cell lines (lower panel). In melanoma cell lines, only microscopy robustly detected growth inhibition by ribociclib, while in neuroblastoma cell lines, both readouts yielded similar results. Red and yellow broken lines indicate the pretreatment signal (CTG and number of cells before addition of ribociclib). Black broken lines indicate cell viability of 0, 0.5, and 1. ATP, adenosine triphosphate; CTG, CellTiter-Glo^®^; DMSO, dimethyl sulfoxide; FITC, fluorescein isothiocyanate.

Interestingly, treatment of melanoma cells with ribociclib led to a visible change in morphology, with cells showing enlargement concomitant with a flattened structure (Figure 2A and Supplementary Figure 4A). A dose-dependent increase in cell area, consistent with an increase in cell size, was detected in all 3 tested melanoma cell lines by microscopy and automated image analysis (Supplementary Figure 4B). In contrast, the neuroblastoma lines showed no visible changes in size under the same conditions (Figure 2A and Supplementary Figure 4A and 4B). We hypothesized that the increased size of G_1_-arrested melanoma cells also led to increased ATP level per cell, thereby producing a stronger CTG signal that masked the reduction in cell numbers. Similar observations have been reported previously [31], and to verify this hypothesis, we directly compared the CTG assay with a microscopy-based assay that directly determined cell numbers from nuclear staining. We obtained markedly different results in the 3 melanoma cell lines (Figure 2B). While CTG indicated virtually no response to ribociclib, cell numbers from microscopy were clearly reduced in a dose-dependent manner. Conversely, we found a high concordance between the assays for the 3 neuroblastoma lines.

We extended our comparison of CTG and microscopy to a panel of 49 cell lines derived from 7 different lineages (Supplementary Table 4). Overall, the assay correlation was poor, with only few cell lines, including the 3 previously described neuroblastoma lines, showing reasonable concordance between the 2 assay formats (Supplementary Figure 4C). Moreover, CTG measurements frequently indicated lower sensitivity than microscopy. Interestingly, this discrepancy was seen in 2 HR-positive breast cancer cell lines, where the direct microscopic cell count measurement was corroborated for both cell lines in the previously described CyQuant assay (Table 1). These data suggest that in profiling the effect of CDK4/6 inhibitors that induce G_1_ block in certain cells, such as ribociclib, using alternative assay formats, including microscopy, may better enable the identification of markers predictive of response. We also cannot rule out the possibility that the association of sensitivity with *RB1* status that was previously observed in our cell line screen (Supplementary Figure 3C) could be confounded by the failure of the CTG assay to accurately report reductions in cell number. However, it is unlikely that this would occur at a greater frequency in *RB1*-deficient cell lines than in *RB1*-proficient cell lines. All cell lines measured by both CTG and microscopy (Supplementary Table 4) were *RB1*-proficient.

### Ribociclib inhibits tumor growth *in vivo*

Mouse knockout studies have indicated that myelosuppression is an anticipated on-target effect of CDK4/6 inhibition [32]. To further characterize the pharmacokinetic/pharmacodynamic (PK/PD) relationship and simultaneously compare antitumor effects with white blood cell (WBC) counts in host animals, we used the JeKo-1 xenograft model in nude rats. We first conducted a PK/PD analysis where ribociclib was administered at 10, 75, and 150 mg/kg once daily for 5 days in JeKo-1 tumor–bearing rats. We observed dose-proportional increases in both drug exposure and target inhibition (Supplementary Table 5). While ribociclib 10 mg/kg daily resulted in partial inhibition (maximally 46%) of Rb phosphorylation in tumor tissue over a 24-hour period after the last dose, >90% inhibition of Rb phosphorylation was observed for most time points at the 75 and 150 mg/kg doses. Notably, at the 150 mg/kg dose, near complete inhibition of Rb phosphorylation was achieved (≥97%) for the entire 24-hour period following ribociclib administration (Supplementary Table 5 and Supplementary Figure 5). This dose-proportional target inhibition correlated with dose-dependent antitumor efficacy in the same JeKo-1 xenograft model.

**Figure 3:**
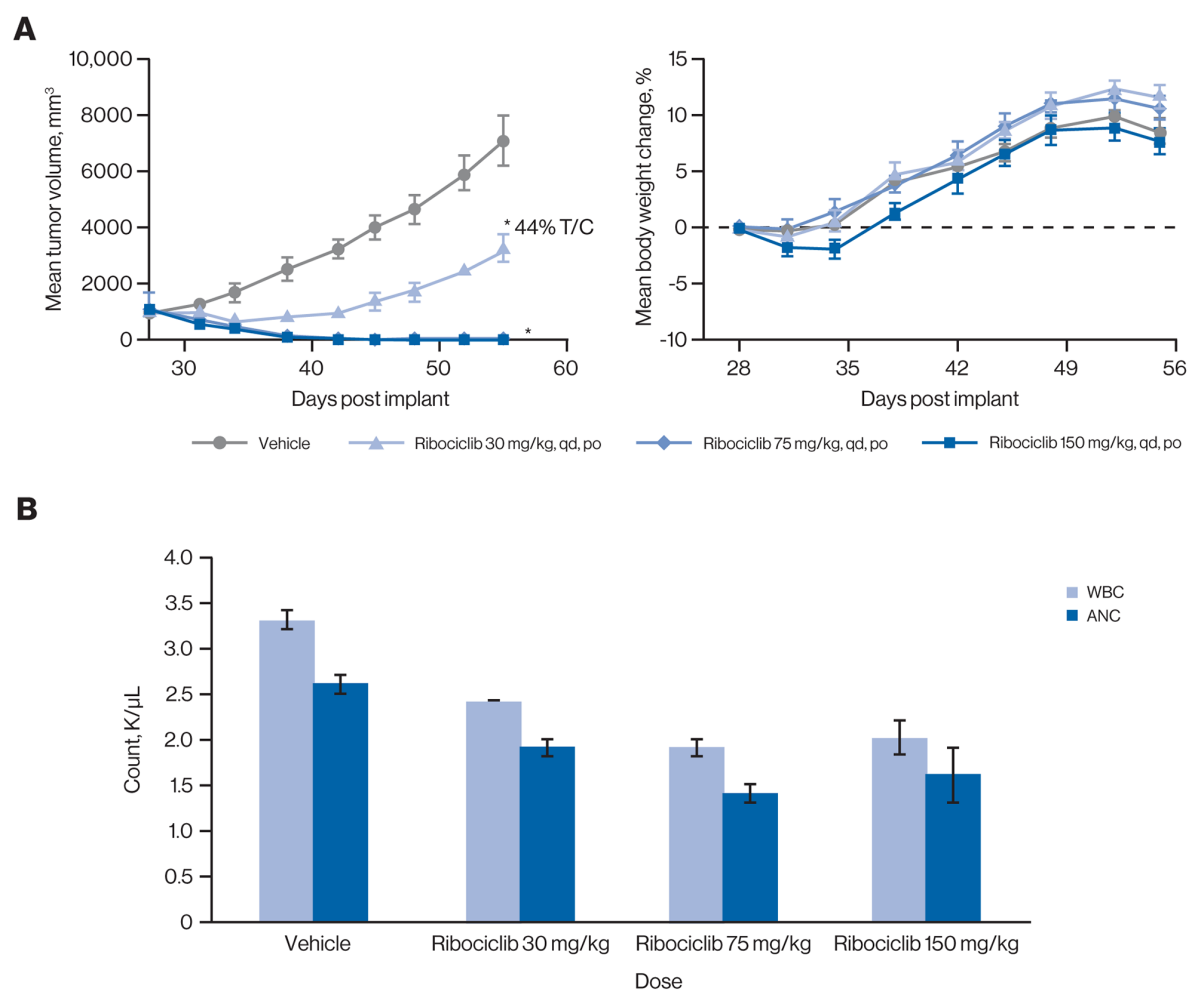
Ribociclib induces robust inhibition of tumor growth *in vivo*, concomitant with on-target myelosuppression **(A)** Ribociclib induced total tumor regressions at the 75 mg/kg and 150 mg/kg doses (left panel) with only minimal transient body weight loss at the 150 mg/kg dose (right panel) in the JeKo-1 rat xenograft model. Error bars show the SEM. ^*^P<0.05 compared with vehicle control (one-way analysis of variance followed by post hoc Tukey [n=7]). **(B)** Dose-dependent reductions of WBCs and ANC were observed in rats treated with ribociclib. Maximum inhibition was seen with the 75 mg/kg and 150 mg/kg doses. Black broken line indicates mean body weight change of 0. Error bars show the SEM. ANC, absolute neutrophil count; po, orally; qd, once daily; SEM, standard error of the mean; T/C, treatment/control; WBC, white blood cell.

We then evaluated antitumor efficacy of the same doses in a separate analysis with longer duration (Figure 3A). The 30 mg/kg dose led to moderate (56%) tumor growth inhibition, while both the 75 and 150 mg/kg doses resulted in complete tumor regression (Figure 3A). All ribociclib doses were well tolerated with no significant body weight loss observed in any treatment group. However, total WBC counts and absolute neutrophil counts were reduced in all treatment groups compared with vehicle (Figure 3B). Together, these data demonstrate that ribociclib induces dose-dependent antitumor activity in a rat model harboring CDK4-dependent tumors while simultaneously generating expected on-target myelosuppression. Ribociclib showed a range of single-agent activity in Rb-proficient tumor models grown as xenografts in mice, while no appreciable antitumor effect was detected in 6 breast cancer patient-derived xenograft (PDX) models in which Rb was undetectable by immunohistochemistry (Supplementary Table 6).

Although the maximum tolerated dosage in mice was determined to be 250 mg/kg once daily, we opted to reduce the dosage in mouse efficacy studies to 75 mg/kg once daily after human PK and tolerability data became available [33] in order to mimic the clinical PK of ribociclib as best as possible. Given that the half-life of ribociclib is substantially shorter in mice than in humans, a perfect dose match is not possible. At 75 mg/kg once daily, the maximum serum concentration in mice (∼2700 ng/mL) is higher than that in humans at 600 mg once daily (∼1700 ng/mL), while the area under the concentration-time curve in mice (∼12,500 ng/mL × h) is lower than that in humans (∼22,000 ng/mL × h; data not shown). Thus, we considered a dose of 75 mg/kg once daily in mice a good compromise to approximate the clinically achievable exposure, given the half-life differences.

### Ribociclib is an effective combination partner *in vivo*

**Figure 4:**
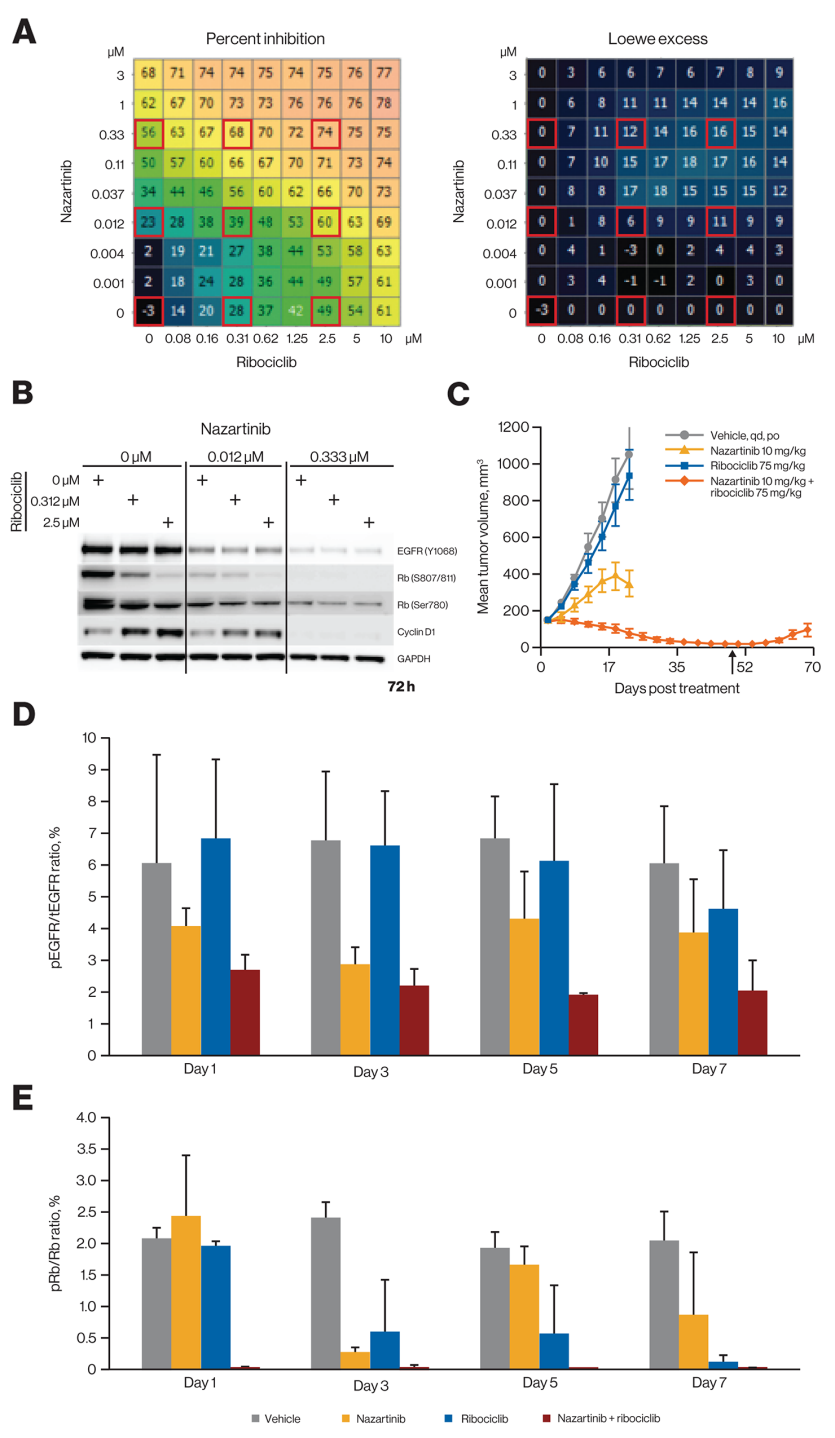
Ribociclib and nazartinib show combinatorial benefit and profound tumor regression in *EGFR*-mutant lung cancer models at a low nazartinib dose **(A)** Treatment of *EGFR*-mutant NCI-H1975 lung cancer cells with nazartinib and ribociclib in a dose-matrix experiment. Growth inhibition and excess inhibition over single-agent treatment according to the Loewe excess model are shown. Calculations have been described previously [34]. **(B)** Western blot analysis of NCI-H1975 cells after treatment as indicated. The respective treatment conditions are also marked by red squares in panel A. Images were collected using the GE Healthcare ImageQuant LAS 4000 imager and software (catalog number: 28955810, version 1.2, build 1.2.1.119) and were cropped, aligned, and annotated using Microsoft PowerPoint. **(C)** Effect of *in vivo* treatment with nazartinib and ribociclib alone and in combination on tumor volume at indicated doses in the *EGFR*-mutant NSCLC cancer PDX model LU1868. Arrow indicates end of treatment. Error bars show the SEM. **(D)** pEGFR/tEGFR ratios for different treatment regimens in PDX model LU1868. Error bars show the SEM. **(E)** pRb/Rb ratios for different treatment regimens in PDX model LU1868. Error bars show the SEM. PDX, patient-derived xenograft; NSCLC, non-small cell lung cancer; pEGFR, phosphorylated epidermal growth factor receptor; pRb, phosphorylated retinoblastoma protein; SEM, standard error of the mean; tEGFR, total epidermal growth factor receptor.

Because CDK4/6, in conjunction with D cyclins, acts downstream of several oncogenic pathways [2, 10], ribociclib might be an effective combination partner with inhibitors that directly target these oncogenic pathways; this concept is supported by recent studies indicating CDK4/6 inhibitors can effectively combine with a variety of targeted therapies [34–39]. To further explore this concept, we examined additional combinations of ribociclib with inhibitors of key oncogenic drivers *in vivo*. EGFR is genetically activated in a substantial population of lung adenocarcinomas [40], and EGFR inhibitors were proven to be highly effective in treating this genetically defined subpopulation of cancer. However, despite excellent initial responses, as with most cancer treatments, durability of responses remains a challenge. We first evaluated the combination of the third-generation EGFR inhibitor nazartinib (EGF816) [41, 42] with ribociclib *in vitro*. Using high-content imaging for automated cell counting, we screened several *EGFR*-mutant cell lines, including the T790M-containing NCI-H1975 cell line (Figure 4A and data not shown), with a 9 by 9 combination matrix of nazartinib versus ribociclib. Varying antiproliferative synergy was observed across these cell lines that corresponded with changes observed in mechanistic readouts, including phospho-EGFR, phospho-Rb, and cyclin D1 levels (Figure 4B). The greatest antiproliferative synergy was observed at concentrations that affected all 3 markers.

This combination was then tested *in vivo* using an *EGFR*-mutant PDX model of non-small cell lung cancer (NSCLC). While single-agent nazartinib at 10 mg/kg once daily was only moderately efficacious, the combination with ribociclib at 75 mg/kg once daily led to sustained tumor regression, although tumors slowly relapsed after discontinuing the treatment (Figure 4C). The combination also led to stronger suppression of both phosphorylated EGFR and Rb in tumors (Figure 4D and 4E). Upon increasing the dose of nazartinib to 30 mg/kg, single-agent activity was sufficient for deep regression, and no combination benefit was observed with addition of ribociclib (Supplementary Figure 6A). Similarly, when treating NCI-H1975 xenografts with nazartinib 10 mg/kg, a combination benefit was apparent when ribociclib was added to the regimen, while nazartinib 30 mg/kg exhibited substantial single-agent antitumor efficacy (Supplementary Figure 6B and 6C). All regimens were well tolerated (data not shown). Thus, addition of ribociclib may lower the required dose of nazartinib needed to achieve optimal efficacy.

**Figure 5:**
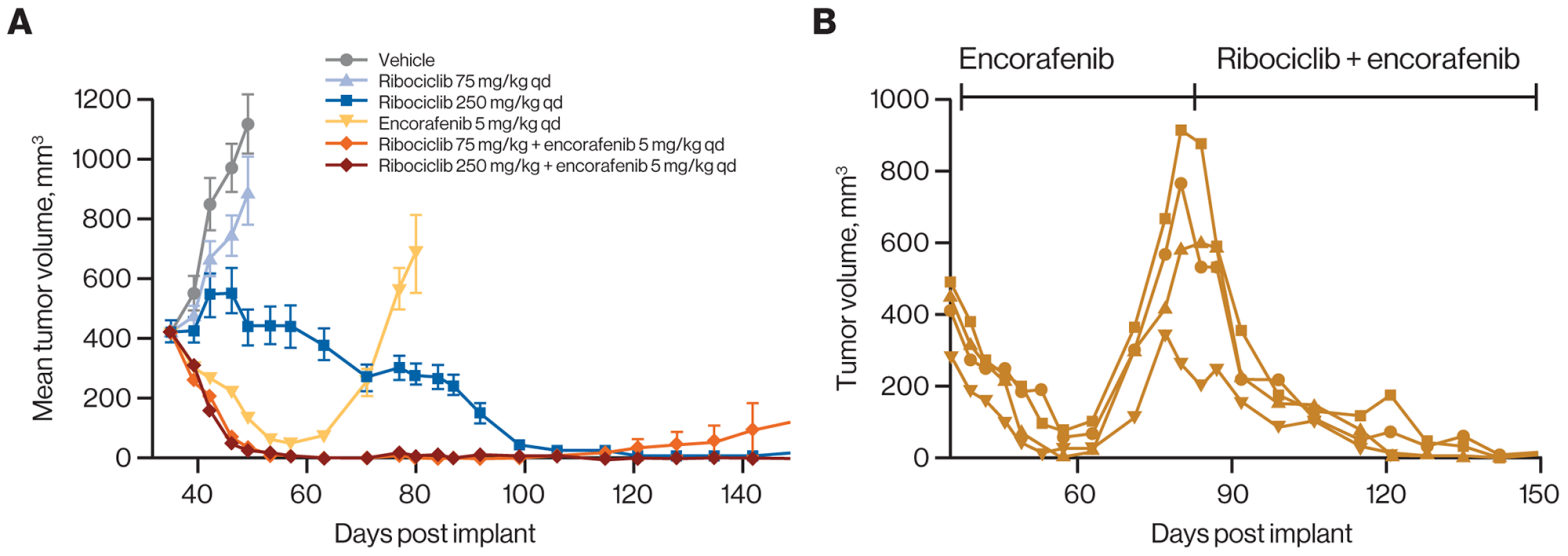
Ribociclib prevents emergence of resistance under treatment when combined with encorafenib in a *BRAF*-mutant melanoma model **(A)** Treatment of HMEX1906 *BRAF* V600E mutant melanoma PDX models with the indicated regimens. Ribociclib showed delayed single-agent activity at 250 mg/kg, but not at 75 mg/kg. As expected, encorafenib induced tumor regression followed by rapid emergence of resistance, while addition of 75 mg/kg of ribociclib prevented the emergence of resistance. Error bars show the SEM. **(B)** Mice (n=4) received daily encorafenib treatment from day 35 to 80 when resistant tumor relapsed on treatment. These mice then received daily combination treatment of 250 mg/kg ribociclib and 5 mg/kg encorafenib for the remaining duration of treatment until day 150. Individual responses are shown. PDX, patient-derived xenograft; qd, once daily; SEM, standard error of the mean.

The T1799A mutant allele of *BRAF* encodes the constitutively active V600E variant form of the kinase. This variant is a key oncogenic driver in multiple cancer types, most notably melanoma [43, 44]. One consequence of *BRAF* V600E expression is an increase in cell proliferation that results, at least in part, from elevated expression of cyclin D1 induced by activated RAF/MEK/ERK signaling. A previous study using a panel of melanoma-derived PDX models harboring the *BRAF* V600E variant described increases in both response rate and duration when ribociclib was combined with the selective *BRAF* inhibitor encorafenib and compared with single agents [34]. These experiments employed ribociclib doses that, while well tolerated, led to higher exposures in mice than are typically achieved in humans at the recommended 600-mg dose [18]. We investigated ribociclib 250 mg/kg and a lower, more clinically relevant dose (75 mg/kg) both alone and in combination with 5 mg/kg of encorafenib in the patient-derived melanoma model HMEX1906 (*BRAF* V600E). Consistent with earlier results, 14 days of treatment with single-agent encorafenib and combined encorafenib and ribociclib resulted in regressions of 69% and 93% (for both 75 and 250 mg/kg ribociclib doses), respectively, while single-agent ribociclib displayed either tumor stasis (250 mg/kg: 1% treatment/control [T/C]) or minimal effects (75 mg/kg: 69% T/C) over this same period of dosing (Figure 5A). With continued treatment, all tumors (8/8) treated with single-agent encorafenib became resistant to treatment within 25 to 45 days and rapidly recurred (Figure 5A). In contrast, when encorafenib was combined with 75 mg/kg and 250 mg/kg of ribociclib, only 2 of 8 and 0 of 8 tumors relapsed while on treatment, respectively. Moreover, recurrence of the 2 tumors treated with encorafenib combined with ribociclib 75 mg/kg was substantially delayed (>80 days) relative to tumors treated with single-agent encorafenib. Interestingly, treatment with high-dose ribociclib resulted in sustained tumor regressions, and no instances of recurrence were detected by the time the experiment was halted. Thus, combining the lower clinically relevant dose of ribociclib, which was ineffective as a single agent, with encorafenib resulted in either a prevention or delay in resistance to encorafenib in all animals treated.

To determine whether encorafenib-resistant tumors are sensitive to ribociclib treatment, 4 HMEX1906 tumors progressing on treatment with encorafenib were rechallenged with combined encorafenib (5 mg/kg) and ribociclib (250 mg/kg) treatment. All treated tumors rapidly regressed with no evidence of relapse observed at the time of treatment termination (Figure 5B). Thus, resistance to encorafenib in the HMEX1906 model did not confer cross-resistance to combined ribociclib and encorafenib treatment.

**Figure 6:**
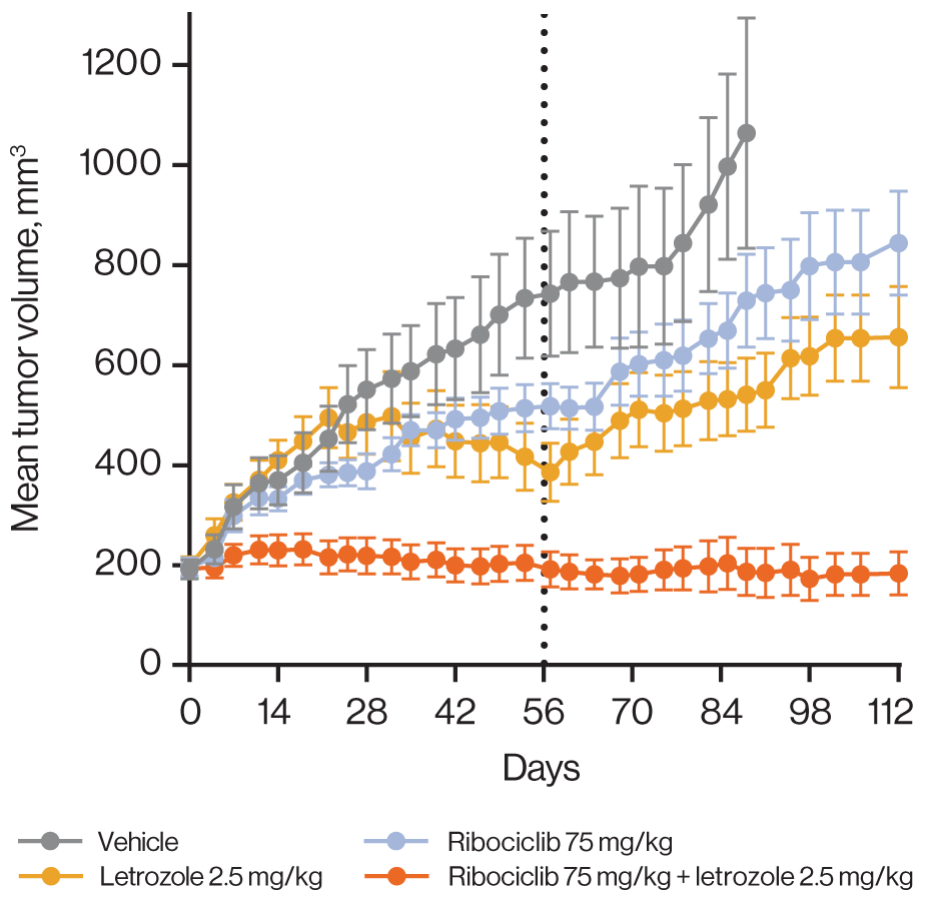
Ribociclib improves response depth when combined with letrozole in a hormone-dependent breast cancer model Combination of ribociclib with letrozole in the HBCx-34 tumor model. Treatment was administered for 56 days (black dotted line). Error bars show the standard error of the mean.

In our PDX clinical trial [33], we observed promising activity of ribociclib in combination with the phosphatidylinositol 3-kinase α inhibitor alpelisib or the mammalian target of rapamycin inhibitor everolimus in breast cancer. Patient-derived xenograft models of diverse HR subtypes were included in this PDX mouse clinical trial, and tumors were grown without hormone supplementation. Efficacy of ribociclib combinations did not stratify with HR status of the PDX models (Supplementary Figures 7 and 8). However, ribociclib and everolimus combination treatment demonstrated a 76% objective response rate, with clear preference for *RB1*-expressing PDX models regardless of HR subtypes (Supplementary Figure 9). Given the previously described sensitivity of HR-positive breast cancer models to CDK4/6 inhibition *in vitro* [11], we tested ribociclib and standard-of-care endocrine therapies in a breast cancer PDX model (HBCx-34) that was grown in the presence of estrogen supplementation and had shown response to endocrine therapies in other studies [45]. Ribociclib and letrozole were tested alone or in combination, revealing markedly enhanced activity with combination therapy over single agents and prolonged stasis even after cessation of treatment (Figure 6 and Supplementary Figure 10). These results lend strong support for combining ribociclib with endocrine therapies in HR-positive breast cancer.

## DISCUSSION

Multiple oncogenic signaling pathways converge on the CDK4/6–cyclin D–Rb axis to promote cell-cycle progression, suggesting selective inhibition of CDK4/6 as an attractive therapeutic strategy. Accordingly, several studies have demonstrated the role of CDK4/6 in solid tumors and hematologic malignancies, as well as the robust antitumor activity associated with CDK4/6 inhibition [3, 10, 12–16]. Currently, 3 CDK4/6 inhibitors (palbociclib, ribociclib, and abemaciclib) are approved. Biochemical analyses indicate that ribociclib is a potent and selective CDK4/6 inhibitor (Brain C et al, manuscript in preparation). KINOME*scan* screening with ribociclib at 0.1 μM revealed exclusive binding to CDK4 (with CDK6 being absent from the screening panel), while at 1 μM, very few kinases exhibited nonspecific binding. This profile was comparable with palbociclib, which showed similar potency as ribociclib. Importantly, and consistent with the biochemical inhibition data, this selectivity extends to closely related family members such as CDK1 and CDK9 [21, 22], which are essential for the proliferation of normal cells [10] and should ideally be unaffected in order to avoid toxicities. While abemaciclib had a higher potency (∼10 fold) compared with the other 2 inhibitors, it exhibited an affinity for additional kinases, even at a concentration as low as 0.1 μM, suggesting a lower degree of selectivity, even when the increased affinity of abemaciclib toward CDK4 was accounted for (Figure 1).

Neutropenia is a dose-limiting toxicity in patients receiving either palbociclib or ribociclib, which is in line with preclinical observations [19]. While neutropenia cases reported with abemaciclib are relatively less frequent, diarrhea is the most common adverse event with this drug [46]. A higher activity of abemaciclib against CDK4 than against CDK6 has been suggested as the reason for reduced neutropenia compared with other CDK4/6 inhibitors [46]. Our data from cell lines with validated CDK4 or CDK6 dependencies confirmed the observation that abemaciclib, unlike palbociclib, shows an increased selectivity for CDK4 versus CDK6. However, in these studies, ribociclib showed an even stronger relative preference for CDK4 versus CDK6 than abemaciclib. Given these results, we do not consider the preferential activity for CDK4 versus CDK6 as a likely explanation for the frequency of neutropenia. Alternatively, the lower selectivity of abemaciclib could lead to off-target dose-limiting toxicities due to diarrhea before sufficient CDK6 inhibition can be achieved, which would subsequently lead to neutropenia.

The antiproliferative activity of therapeutic agents in preclinical studies is routinely measured using assay principles that rely on the metabolic activity of viable cells, particularly in high throughput setups. In our studies, we made the unanticipated observation that effects of CDK4/6 inhibition are not adequately captured using an ATP-based viability assay, which was likely because of cell enlargement and ATP accumulation upon G_1_ cell-cycle arrest. Hence, cell counting (by microscopy or other methods) appears to be a more accurate readout that enables consistent appreciation of the antiproliferative effects of CDK4/6 inhibitors across all tested cell lines and may ultimately be a better predictor of *in vivo* efficacy. Interestingly, we previously noted that combination effects of ribociclib are more pronounced *in vivo* than *in vitro* [34], which in hindsight may have been an underestimation of the *in vitro* effects because of the viability assay used. A striking discrepancy between ATP quantification and microscopy was observed in 2 HR-positive breast cancer cell lines. In agreement with the more pronounced effects detected by microscopy, those cell lines also displayed clear CDK4 knockdown effects in our pooled shRNA screening data, which was also based on counting cells. In general, our observations argue for a careful interpretation of proliferation data and choice of assay when investigating agents that primarily act by inducing cell-cycle arrest.

Ribociclib has shown single-agent activity in preclinical *in vivo* models [6] and in patients [33], but its ability to improve antitumor efficacy of other agents in combination and/or combat the emergence of treatment resistance [6] adds a significant therapeutic value. A large-scale “mouse clinical trial” campaign using PDX models [34] had uncovered the potential of ribociclib as a versatile combination partner, and data presented here further highlight the ability of ribociclib combinations to improve response depth and delay the emergence of resistance (Figures 4 and 5, Supplementary Figures 5-7). Additionally, in clinical trials, ribociclib has demonstrated robust activity in combination with endocrine therapies for the treatment of estrogen receptor–positive breast cancer [19, 20] (Figure 4 and 5).

In conclusion, we have demonstrated through our *in vitro* and *in vivo* studies that ribociclib is a highly selective CDK4/6 inhibitor that causes the G_1_ arrest of tumor cells containing intact Rb. Ribociclib was efficacious and well tolerated in mouse models, and our results suggest that robust efficacy can be achieved by combining ribociclib with other antitumor agents. Ribociclib is approved in combination with an aromatase inhibitor as initial endocrine-based therapy for the treatment of postmenopausal women with HR-positive, HER2-negative advanced or metastatic breast cancer and is currently being evaluated in several additional clinical trials.

## MATERIALS AND METHODS

### Cell lines and compounds

All compounds were synthesized in-house by Novartis. All cell lines used in this manuscript were obtained from commercial sources as described in Barretina et al [27].

### Kinase selectivity panel and CDK4/6 enzyme assay

Kinase reactions were carried out in 384-well microtiter plates (30 μL per reaction) in 1× assay buffer (50 mM HEPES-Na, pH 7.5, 10 mM MgCl_2_, 1 mM dithiothreitol, 0.02% Tween^®^ 20, 0.05% bovine serum albumin) and analyzed with the Dako EnVision^®^ Detection System. Additional information can be found in the Supplementary Methods. CDK4/6 inhibitors were subjected to the DiscoveRx KINOME*scan* selectivity screen [47]. Images were generated using the TREE *spot*™ software tool and reprinted with permission from KINOME*scan*. Kinases that bind were marked with red circles if <35% of the respective recombinant kinase was captured on the immobilized ligand in the presence of the indicated concentration of CDK4/6 inhibitor, normalized to a dimethyl sulfoxide control. Larger circles indicate a higher affinity of binding. Binding of 468 kinases (excluding CDK6) or their mutant variants at different concentrations by ribociclib, palbociclib, and abemaciclib was tested.

### *In vitro* viability assays for ribociclib

To determine the effects of ribociclib on cell proliferation *in vitro*, 750 to 1500 cells per well were seeded in 384-well plates. Ribociclib was tested at 8 dose points and 1:3 dilution steps from 4.5 nM to 10 μM. After 72 hours of treatment for ≥3 replicate wells, single-agent effects were assessed by both quantification of cellular adenosine triphosphate levels by CTG or by microscopy using an IN Cell Analyzer 2000 (GE Healthcare) equipped with a 4× objective and DAPI excitation/emission filters and a CCD camera. To assess cell size, 4 images per well were captured with a 10× objective using DAPI (for Hoechst 33342) and fluorescein isothiocyanate (for anti–α-tubulin) excitation/emission filters. Images were acquired using the IN Cell Analyzer 2000 software and analyzed using adapted methods described in [36] and using the R/Bioconductor package EBImage [48].

### *In vivo* studies

### Ethics statement

Animal studies comply with Novartis Global Standards and Principles for the Care and Use of Animals, in accordance with National Research Council 2011 standards and are conducted under protocols approved by Institutional Animal Care and Use Committee (protocol #12-ONC-016).

Animals were housed in a temperature- and humidity-controlled vivarium with a 12-hour light/dark cycle and were provided with food and water ad libitum. Tumor volumes were measured regularly by caliper and calculated using the ellipsoid formula ([length × width^2^]/2). Body weight data are presented as percent change in body weight from the day of treatment initiation and expressed as mean ± standard error of the mean.

### Pharmacokinetic, pharmacodynamic, and efficacy studies in the JeKo-1 rat model

For PK, PD, and efficacy studies in the mantle cell lymphoma model, male nude rats (NTac:NIH-*Foxn1*^*rnu*^ [Taconic]) were sublethally irradiated, and then JeKo-1 cells with Matrigel™ (BD Biosciences) supplementation were implanted subcutaneously. Rats bearing established tumors received oral ribociclib daily at various dosages to evaluate dose-dependent changes in drug exposure, Rb phosphorylation, and tumor growth effects. Additional information can be found in the Supplementary Methods.

### Tumor growth efficacy studies with combination of ribociclib and targeted therapies

For evaluation of ribociclib plus nazartinib in *EGFR*-mutant NSCLC, female athymic nude mice (Crl:NU[Ncr]-*Foxn1*^*nu*^ [Charles River Laboratories]) were implanted subcutaneously with LU1888 primary NSCLC xenograft. Mice bearing established tumors received oral ribociclib and nazartinib daily, as single agents and combinations, at clinically relevant dosages.

For evaluation of ribociclib plus encorafenib in *BRAF*-mutant melanoma, female athymic nude mice were implanted subcutaneously with HMEX1906 primary melanoma xenograft. Mice bearing established tumors received oral ribociclib and encorafenib daily, as single agents and combinations, at clinically relevant dosages. Mice received continuous daily drug treatments until tumors relapsed (tumor volume >1000 mm^3^) to investigate treatment effects on resistance development. Further details of these studies are provided in the Supplementary Methods.

## SUPPLEMENTARY MATERIALS FIGURES AND TABLES




