# Correction: The potent and selective cyclin-dependent kinases 4 and 6 inhibitor ribociclib (LEE011) is a versatile combination partner in preclinical cancer models

**DOI:** 10.18632/oncotarget.27407

**Published:** 2020-04-07

**Authors:** Sunkyu Kim, Ralph Tiedt, Alice Loo, Thomas Horn, Scott Delach, Steven Kovats, Kristy Haas, Barbara Schacher Engstler, Alexander Cao, Maria Pinzon-Ortiz, Iain Mulford, Michael G. Acker, Rajiv Chopra, Christopher Brain, Emmanuelle di Tomaso, William R. Sellers, Giordano Caponigro

**Affiliations:** ^1^ Novartis Institutes for BioMedical Research, Oncology Disease Area, Cambridge, MA, USA; ^2^ Novartis Institutes for BioMedical Research, Oncology Disease Area, Basel, Switzerland, USA; ^3^ Novartis Institutes for BioMedical Research, Chemical Biology & Therapeutics, Cambridge, MA, USA; ^4^ Novartis Institutes for BioMedical Research, Global Discovery Chemistry, Cambridge, MA, USA

**This article has been corrected:** In Table 1, a unit of measurement is displayed incorrectly. “μM” is used instead of “nM”. The corrected Table 1 is shown below. The authors declare that these corrections do not change the results or conclusions of this paper.

Original article: Oncotarget. 2018; 9:35226–35240. 35226-35240. https://doi.org/10.18632/oncotarget.26215

**Table 1:** IC_50 _Values of CDK4/6 Inhibitors

Cell line	Cancer type	Dominant CDK	Ribociclib IC_50_, mean ± SD, nM	Palbociclib IC_50_, mean ± SD, nM	Abemaciclib IC_50_, mean ± SD, nM
JeKo-1	MCL	CDK4	143 ± 87	72 ± 33	20 ± 9
CAMA-1	ER+ BC	CDK4	162 ± 59	50 ± 24	28 ± 2
MCF-7	ER+ BC	CDK4	62 ± 30	30 ± 18	11 ± 7
T47D	ER+ BC	CDK4	111 ± 14	66 ± 19	13 ± 3
REH	ALL	CDK6	1030 ± 246	60 ± 17	72 ± 6
SEM	ALL	CDK6	1484±215	87 ± 28	162 ± 37
Pfeiffer	DLBCL	CDK6	948 ± 53	89 ± 32	66 ± 25
MOLM-13	AML	CDK6	365 ± 62	47 ± 25	57 ± 21

IC_50_values (mean ± SD) of ribociclib, palbociclib, and abemaciclib were determined using the CyQuant cell proliferation assay. The average differential for CDK4 versus CDK6 dependent lines for ribociclib, palbociclib, and abemaciclib is 8.0-, 1.3-, and 5.5-fold, respectively. Abbreviations: ALL, acute lymphoblastic leukemia; AML, acute myeloid leukemia; BC, breast cancer; CDK, cyclin-dependent kinase; DLBCL, diffuse large B-cell lymphoma; ER+, estrogen receptor-positive; IC_50_, half-maximal inhibitory concentration; MCL, mantle-cell lymphoma; SD, standard deviation.

