# Supplementary material for: The potent and selective cyclin-dependent kinases 4 and 6 inhibitor ribociclib (LEE011) is a versatile combination partner in preclinical cancer models
Source: Oncotarget. 2018 Oct 16;9(81):35226–40. doi: 10.18632/oncotarget.26215 (PMC6219668; doi:10.18632/oncotarget.26215)
Supplement: Supplementary file 1 [file oncotarget-09-35226-s001.pdf]

The potent and selective cyclin-dependent kinases 4 and 6 inhibitor ribociclib (LEE011) is a versatile combination partner in preclinical cancer models

SUPPLEMENTARY METHODS

CDK4/6 enzyme assay

Kinase reactions were carried out in 384-well microtiter plates (30 µL per reaction) in 1× assay buffer (50 mM HEPES-Na, pH 7.5, 10 mM $MgCl_2$, 1 mM dithiothreitol, 0.02% Tween® 20, and 0.05% bovine serum albumin [BSA]). The reaction protocol/conditions for the cyclin-dependent kinase 4 (CDK4)–cyclin D1 assay were as follows: 2 µL of test compound in 20% dimethyl sulfoxide (DMSO) was added to 18 µL of 0.5 nM CDK4-cyclin D1, and then 10 µL of a mixture of 450 nM phosphorylated retinoblastoma protein (pRb; 152-end [C-terminus fragment containing CDK4/6 phosphorylation sites]) and 9 µM adenosine triphosphate (ATP) was added. The reaction protocol/conditions for the CDK6-cyclin D3 assay were as follows: 2 µL of test compound in 20% DMSO was added to 18 µL of 0.84 nM CDK6-cyclin D3, and then 10 µL of a mixture of 450 nM pRb (152-end) and 9 µM ATP was added. The reactions were incubated at 22°C for 60 and 120 minutes for CDK4-cyclin D1 and CDK6-cyclin D3, respectively, after which 10 µL 160 mM ethylenediaminetetraacetic acid (EDTA)-Na (pH 8.0) was added to stop the reaction. For detection of activity, 40 µL of a solution containing 1× detection buffer (50 mM HEPES-Na, pH 7.5, 60 mM EDTA-Na, pH 8.0, 0.05% BSA, and 0.1% Triton X-100® [MP Biomedicals; catalog number: 195458]), 160 nM allophycocyanin-streptavidin, 4 nM Eu-W1024 anti-rabbit immunoglobulin G antibody, and 286 ng/mL phospho-Rb (Ser780) antibody (CST) was added to the reaction mixtures and incubated at 22°C without light for 60 minutes before analysis with the Dako EnVision® Detection System.

Enzyme-linked immunosorbent assay to quantitate Rb phosphorylation

4H1 Rb antibody #9309 (CST; catalog number: 9309) was added to clear MaxiSorp® plates (ThermoScientific) at a 25 ng/well concentration in 50 µL of Dulbecco's Phosphate Buffered Saline (Gibco; catalog number: 14190-144) and incubated overnight with rocking at 4°C. After washing with Tris buffered saline solution–Tween 20 (TBST; Teknova; catalog number: T9501), 250 µL of SuperBlock® Blocking Buffer (Pierce; catalog number: 37535) was added, and the mixture was incubated for 1 hour. After blocking, 10 µL of cell lysates containing 10 µg of total protein were added in triplicate wells; 40 µL of phosphate buffered saline (PBS; Gibco; catalog number: 10010-023) containing 10% SuperBlock Blocking Buffer was added to the wells to make a final volume of 50 µL. Plates were sealed, incubated for 2 hours at room temperature, and then washed 3 times with TBST. Then, 50 µL of a 1:1000 dilution of Phospho-Rb (Ser780; catalog number: 9307) antibody in PBS/10% SuperBlock Blocking Buffer was added, and the mixture was incubated overnight at 4°C. Plates were washed 3 times in TBST, then 50 µL of 1:2500 dilution of donkey-anti-rabbit HRP (Amersham; catalog number: NA934V) in PBS/10% SuperBlock Blocking Buffer was added, and the plates were shaken for 30 minutes at room temperature. After washing, 100 µL of 1-Step™ Ultra-TMB enzyme-linked immunosorbent assay (Pierce; catalog number: 34028) was added, and the mixture was incubated for 30 minutes in the dark. Sulfuric acid (100 µL of 2 M) was added to the plate to stop the reaction, and absorbance was read on a SpectraMax® reader (Molecular Devices) at 450 nM within 2 hours.

Fluorescence-activated cell sorting

To identify the distribution of cell-cycle phases in treated cells, we measured their DNA content by fluorescence-activated cell sorting or flow cytometry. Analysis of cellular DNA content by propidium iodide (PI; MP Biomedicals) staining can discriminate between cells that are in the G_0/G_1, S, or G_2/M phase. Cells were transferred to a V-bottomed, 96-well polypropylene plate (using trypsin to remove adherent cells, if necessary) and centrifuged to remove the supernatant media, then 100 µL of a hypotonic lysis buffer (0.1% sodium citrate [Sigma; catalog number: S-4641], 0.1% Triton X-100, 25 µg/mL of PI, and 10 µg/mL of RNAse [Roche; catalog number: 1 579 681]) was added to the cells and incubated at room temperature in the dark for 1 hour and stored at 4°C overnight, if necessary. Last, DNA content was analyzed using the BD™ LSR II System and FACSDiva™, version 5.0.1 (BD biosciences) and ModFit LT™ 3.1 (Verity Software House, Inc).

In vitro viability assays for Ribociclib by CellTiter-Glo® or microscopy

To determine the effects of ribociclib on cell proliferation *in vitro*, 750 to 1500 cells per well were seeded in 80 µL of medium in 384-well plates, briefly centrifuged to promote an even distribution of cells across

the entire well and incubated at room temperature for 30 minutes. All plates were incubated at 37°C in 5% CO_2 for 24 hours before addition of the compound. Then, a 400× compound stock was added using a Peak Analysis & Automation robot equipped with a 200-nL pin tool to achieve a 1× compound concentration. Ribociclib was tested at 8 dose points and 1:3 dilution steps from 4.5 nM and 10 μM. After 72 hours of treatment for a minimum of 3 replicate wells, single-agent effects were assessed by both quantification of cellular ATP levels by CellTiter-Glo (Promega; catalog number: G7573), as described in the manufacturer's protocol, or by microscopy. For quantification by microscopy, cells were fixed and permeabilized for 45 minutes in 4% paraformaldehyde (Electron Microscopy Sciences; catalog number: 15714) and 0.12% TX-100 (Electron Microscopy Sciences; catalog number: 22140) in PBS. After washing the cells 3 times with PBS, DNA and tubulin were stained for 30 minutes with Hoechst 33342 (ThermoFisher; catalog number: H3570) at a final concentration of 4 μg/mL and anti–α-tubulin fluorescein isothiocyanate (FITC) antibody (Sigma; catalog number: F2168) at a final dilution of 1:2500, respectively. Cells were washed again 3 times with PBS, and then the plates were heat-sealed and stored at 4°C until imaging. To quantify cell numbers (equivalent to the number of nuclei revealed by the Hoechst signal), all cells per well were captured in a single image by fluorescence microscopy using an IN Cell Analyzer 2000 (GE Healthcare) equipped with a 4× objective and DAPI excitation/emission filters and a CCD camera. To assess cell size, 4 images per well were captured with a 10× objective using DAPI (for Hoechst 33342) and FITC (for anti–α-tubulin) excitation/emission filters.

Image analysis for Ribociclib *in vitro* effects

Images were acquired using the IN Cell Analyzer 2000 software and analyzed using adapted methods [1] and using the R/Bioconductor package EBImage [2]. Images captured with the 4× objective lens in the DAPI channel (for Hoechst/DNA) were segmented by adaptive thresholding and counted. By analyzing 17 additional object/nuclei features in the DNA channel (ie, shape and intensity features), debris/fragmented nuclei were identified. The distributions of the additional features between staurosporine-positive controls and DMSO-negative controls were compared manually. Features that could differentiate between the conditions (eg, a shift in the distribution of a feature measurement comparing DMSO with staurosporine) were used to define the "debris" population and subtract it from raw nuclei counts to obtain measurements of cell proliferation (ie, cell count). Images captured with the 10× objective lens in the DAPI channel (for Hoechst/DNA) were segmented by adaptive thresholding, and the cell size was estimated from fluorescence levels in the α-tubulin channel by extension from the nuclei in the DAPI channel using a propagation algorithm [3].

In vivo studies

Animal studies comply with Novartis Global Standards and Principles for the Care and Use of Animals, in accordance with National Research Council 2011 standards and are conducted under protocols approved by Institutional Animal Care and Use Committee (protocol #12-ONC-016). Animals were housed in a temperature- and humidity-controlled vivarium with a 12-hour light/dark cycle and were provided with food and water ad libitum. In all studies, tumor volume was calculated using the following formula: (length × width^2)/2. Percent treatment/control (T/C) values were calculated using the following formula: $\%T/C = 100 \times \Delta T/\Delta C$, if $\Delta T > 0$. Percent regression was calculated using the following formula: $\%Reg = 100 \times \Delta T/T_{initial}$, if $\Delta T < 0$. In all equations, T = mean tumor volume of the drug-treated group on the final day of the study; ΔT = mean tumor volume of the drug-treated group on the final day of the study − mean tumor volume of the drug-treated group on initial day of dosing; $T_{initial}$ = mean tumor volume of the drug-treated group on initial day of dosing; C = mean tumor volume of the control group on the final day of the study; and ΔC = mean tumor volume of the control group on the final day of the study − mean tumor volume of the control group on the initial day of dosing. The percent change in body weight (BW) was calculated as $(BW_{current} - BW_{initial})/BW_{initial} \times 100$. Data are presented as the percent change in BW from the day of treatment initiation and expressed as mean ± standard error of the mean.

Pharmacokinetic, pharmacodynamic, and efficacy studies in the JeKo-1 rat model

For pharmacokinetic (PK), pharmacodynamic (PD), and efficacy studies in the mantle cell lymphoma model, male nude rats (NTac:NIH-*Foxn1^{rnu}* [Taconic]) were sublethally irradiated, and then JeKo-1 cells with Matrigel™ (BD Biosciences) supplementation were implanted subcutaneously. Rats bearing tumors ≥800 mm^3 were randomized into treatment groups for daily oral treatment with ribociclib. For the PK/PD study, rats received 5 days of once-daily ribociclib, and then tumors were harvested at designated time points for measurement of Rb phosphorylation by enzyme-linked immunosorbent assay as described previously. Blood was also collected to measure drug concentrations in plasma. For the efficacy study, rats received once-daily ribociclib for 28 days, and tumor volumes were measured regularly to determine the effects of the treatment on tumor growth.

Combination of Ribociclib with Encorafenib

The HMEX1906 xenograft model was confirmed to carry the *BRAF* V600E mutation and deletion of *CDKN2A* (p16; data not shown). Female athymic nude

mice (Crl:NU[Ncr]-*Foxn1^{nu}* [Charles River Laboratories]) were implanted subcutaneously with 100 μL of minced HMEX1906 tumor (passage 6) suspended in 50% Matrigel in the right dorsal axillary region. Treatment for the tumor growth efficacy experiment was initiated when the tumor volume reached an average size of 420 mm^3 (range, 252–642 mm^3, 35 days post tumor implantation). The mice were sorted into the treatment groups shown in Figure 6A (n=8 per group). Ribociclib was administered at doses of 250 mg/kg and 75 mg/kg once-daily, using ribociclib-succinate (salt, powder form) formulated in 0.5% methylcellulose. Encorafenib formulated in 0.5% carboxymethylcellulose and 0.5% Tween 80 was administered at 5 mg/kg twice daily. Body weight and tumor volume were recorded twice weekly. The mice received continuous daily drug treatments until tumors relapsed (tumor volume >1000 mm^3).

Combination of Ribociclib with Letrozole

Tumor fragments of the HBCx-34 patient-derived xenograft model were implanted subcutaneously in 5-week-old female athymic nude mice Crl:NU(Ncr)-*Foxn1^{nu}* (Charles River Laboratories) weighing 18 to 25 g. To stimulate initial rate of intake and tumor growth, HBCx-34 tumor–bearing mice received supplementary estrogen diluted in drinking water (β-estradiol, 8.5 mg/L) from the date of tumor implant to the date of inclusion (ie, for 37 days). Supplementary estrogen was then removed during the drug treatment period. Drug treatment for the tumor growth efficacy experiment was initiated 37 days after implantation of the tumor. Ribociclib was administered at 75 mg/kg in combination with 2.5 mg/kg letrozole for 56 days. Tumor volumes and BWs were evaluated biweekly during the treatment period.

Breast cancer subtype determination in patient-derived xenograft models

Patient-derived xenograft samples were evaluated for HER2 (ERBB2), estrogen receptor (ER; ESR1), and progesterone receptor (PR; PGR) expression. HER2 status was determined by quantifying protein expression with immunohistochemistry (IHC; antibody: Dako polyclonal rabbit antibody [A0485]) and by quantifying gene copy number using Affymetrix™ Genome-Wide Human Single Nucleotide Polymorphism 6.0 Arrays. In addition, expression of ER (antibody: Spring Bioscience, monoclonal rabbit antibody [M3014]) and PR (antibody: Spring Bioscience, monoclonal rabbit antibody [M3024]) were quantified by IHC. Xenograft tumors were determined to be HER2 positive if either of the following was observed: (1) uniform intense membranous staining observed by IHC in at least 30% of cells (3+), or (2) complete membranous staining, either uniform or weak, in at least 10% of cells (2+) and gene copy number greater than 10. Xenograft tumors were determined to be ER positive or PR positive if as few as 1% of tumor cells showed weak immunostaining by IHC. Patient-derived xenograft models were then assigned to 1 of 4 categories: (1) HER2 positive (xenograft tumors scored as HER2 positive regardless of ER and PR status), (2) ER positive (xenograft tumors scored as HER2 equivocal or negative that were ER positive regardless of PR status), (3) PR positive (xenograft tumors scored as HER2 equivocal or negative that were ER negative and PR positive), or (4) triple negative (xenograft tumors scored as HER2 equivocal or negative that were ER and PR negative).

REFERENCES

1. Horn T, Ferretti S, Ebel N, Tam A, Ho S, Harbinski F, Farsidjani A, Zubrowski M, Sellers WR, Schlegel R, Porter D, Morris E, Wuerthner J, et al. High-order drug combinations are required to effectively kill colorectal cancer cells. Cancer Res. 2016; 76:6950–63. https:\\doi.org\10.1158/0008-5472.CAN-15-3425.
2. Pau G, Fuchs F, Sklyar O, Boutros M, Huber W. EBImage--an R package for image processing with applications to cellular phenotypes. Bioinformatics. 2010; 26:979–81. https://doi.org/10.1093/bioinformatics/btq046.
3. Jones T, Carpenter A, Golland P. Voronoi-based segmentation of cells on image manifolds. Comp Vis Biomed Image Appl. 2005; 3765:535–43. https://doi.org/10.1007/11569541_54.
4. McDonald ER 3rd, de Weck A, Schlabach MR, Billy E, Mavrakis KJ, Hoffman GR, Belur D, Castelletti D, Frias E, Gampa K, Golji J, Kao I, Li L, et al. Project DRIVE: a compendium of cancer dependencies and synthetic lethal relationships uncovered by large-scale, deep RNAi screening. Cell. 2017; 170:577–592.e10. https://doi.org/10.1016/j.cell.2017.07.005.
5. Shao DD, Tsherniak A, Gopal S, Weir BA, Tamayo P, Stransky N, Schumacher SE, Zack TI, Beroukhim R, Garraway LA, Margolin AA, Root DE, Hahn WC, et al. ATARiS: computational quantification of gene suppression phenotypes from multisample RNAi screens. Genome Res. 2013; 23:665–78. https://doi.org/10.1101/gr.143586.112.
6. Barretina J, Caponigro G, Stransky N, Venkatesan K, Margolin AA, Kim S, Wilson CJ, Lehar J, Kryukov GV, Sonkin D, Reddy A, Liu M, Murray L, et al. The cancer cell line encyclopedia enables predictive modelling of anticancer drug sensitivity. Nature. 2012; 483:603–7. https://doi.org/10.1038/nature11003.
7. Gao H, Korn JM, Ferretti S, Monahan JE, Wang Y, Singh M, Zhang C, Schnell C, Yang G, Zhang Y, Balbin OA, Barbe S, Cai H, et al. High-throughput screening using patient-derived tumor xenografts to predict clinical trial drug response. Nat Med. 2015; 21:1318–25. https://doi.org/10.1038/nm.3954.

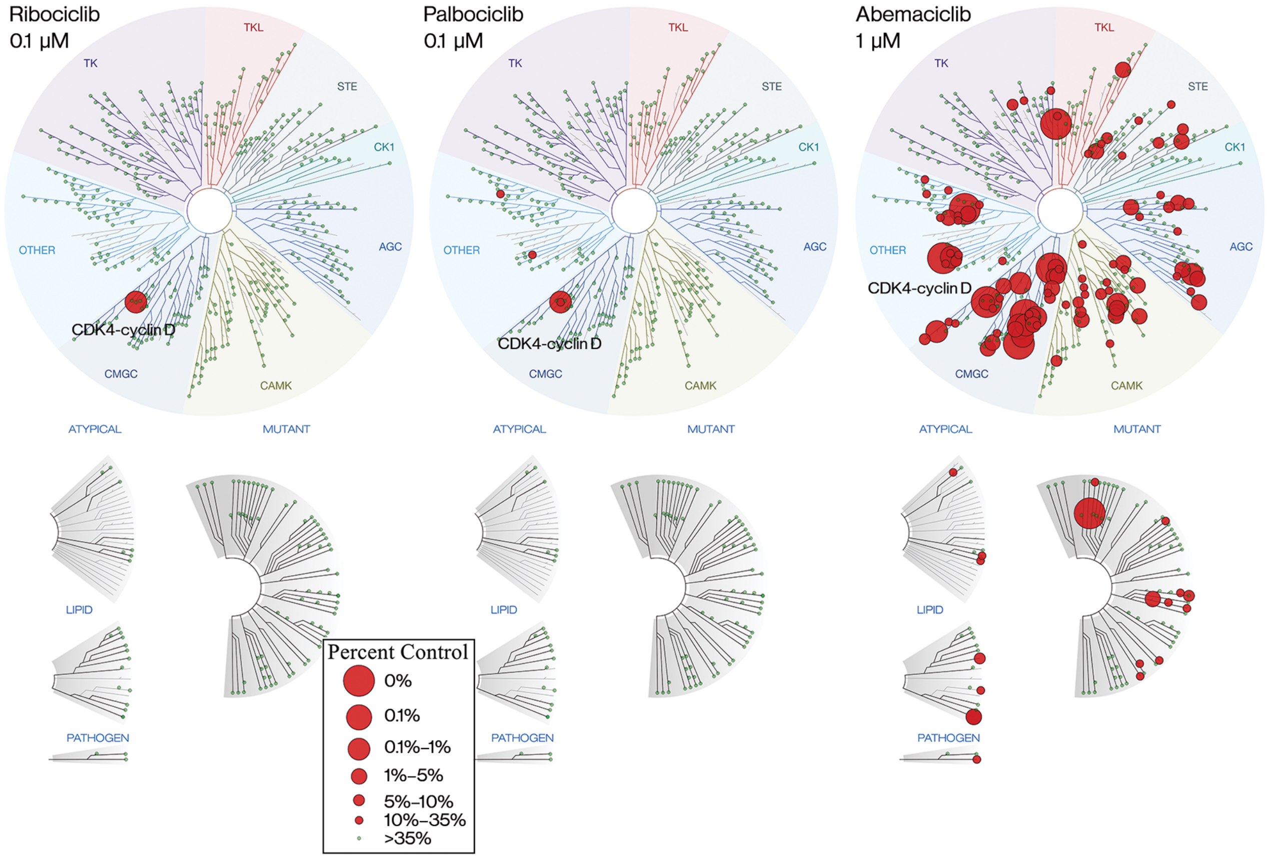

Supplementary Figure 1: Kinase selectivity profiles of ribociclib, palbociclib, and abemaciclib. TREE*spot* view of KINOME*scan* selectivity panel for ribociclib, palbociclib, or abemaciclib at the indicated concentrations. AGC, cAMP-dependent, cGMP-dependent, and protein kinase C; CDK, cyclin-dependent kinase; CK, creatine kinase; CMGC, cyclin-dependent, mitogen-activated glycogen synthase and CDK-like kinase; DMSO, dimethyl sulfoxide; STE, yeast sterile kinase; TK, thymidine kinase; TKL, tyrosine kinase-like.

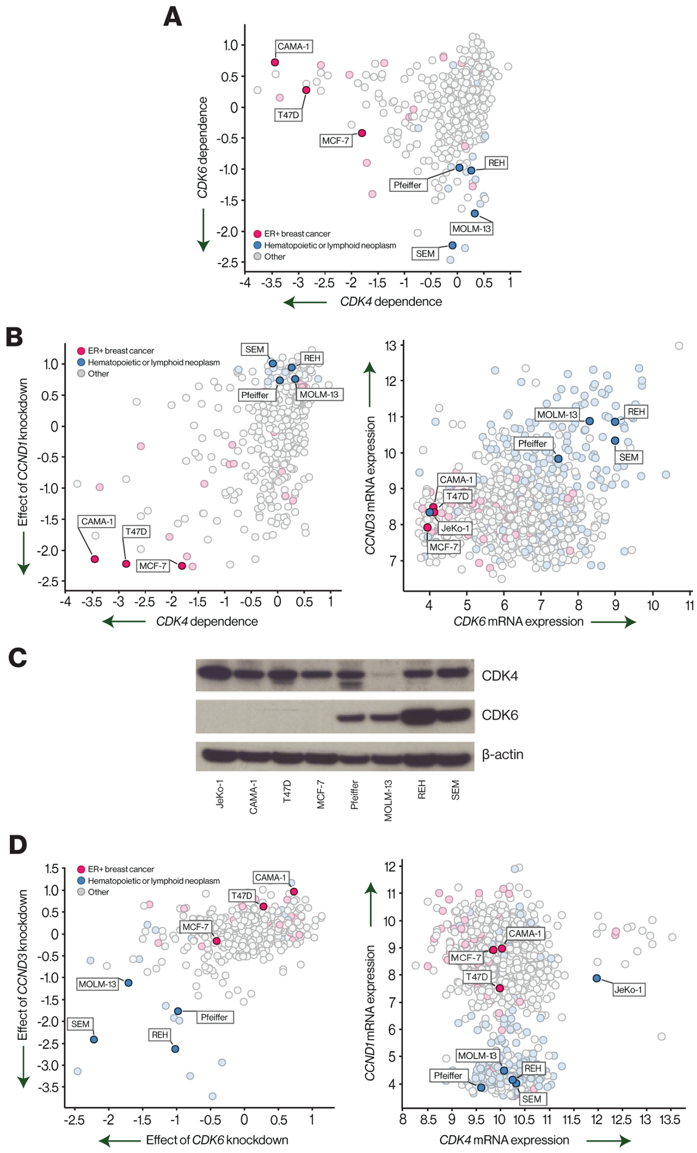

Supplementary Figure 2: Identification of cancer cell lines in which either CDK4 or CDK6 is dominant. **(A)** Effect of CDK4 and CDK6 dependence in 398 cancer cell lines and gene-level ATARiS scores as defined in McDonald et al [4]. There are no data for JeKo-1. **(B)** CDK4-dependent cell lines also tend to depend on CCND1 (left panel; there are no data for JeKo-1). Such cell lines express very low levels of CDK6 mRNA (right panel). **(C)** Confirmation of low/absent CDK6 expression on the protein level in four CDK4-dependent cancer cell lines as opposed to four CDK6-dependent cancer cell lines by Western blot displayed in a cropped format. Images were scanned using an Epson Perfection V600 photo scanner and were processed using Microsoft Office Picture Manager. **(D)** Effect of CDK6 and CCND3 knockdown (left panel; there are no data for JeKo-1). CDK6-dependent cell lines expressed average levels of CDK4 compared with other cell lines, but low levels of CCND1 (right panel). Raw data are also shown in Supplementary Table 2. CDK4/6, cyclin-dependent kinases 4 and 6; mRNA, messenger RNA.

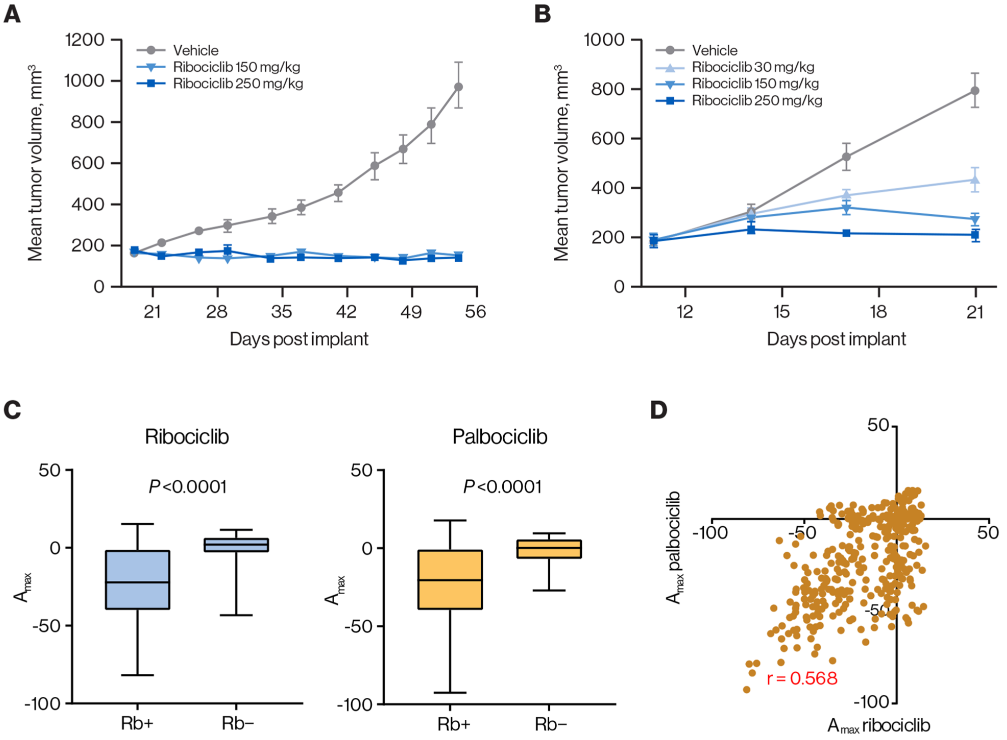

Supplementary Figure 3: Single-agent activity of ribociclib across different cancer cell lines *in vitro* or as mouse xenografts. (A) Antitumor activity of ribociclib against a neuroblastoma xenograft model (CHP-212). Error bars show the SEM. **(B)** Antitumor activity of ribociclib against a malignant rhabdoid tumor xenograft model (G-401). Error bars show the SEM. **(C)** Standard boxplots showing comparison of maximal activity of ribociclib or palbociclib across a large panel of cancer cell lines, stratified by Rb positive (Rb+) or negative (Rb−) status. Rb status was determined considering *RB1* copy number and messenger RNA expression as well as presence of inactivating *RB1* mutations. Sensitivity data were obtained in 3-day proliferation assays with CellTiter-Glo® readout (ribociclib [left box plot]: Rb+ n=443 and Rb− n=28; palbociblib [right box plot]: Rb+ n=340 and Rb− n=23). Each box shows the 25th to 75th percentiles. Lines inside the boxes show the median. Lines outside the boxes represent the minimum and maximum. **(D)** Correlation of maximal effect levels (A_{max}) for ribociclib and palbociclib in 372 cancer cell lines. Data are also shown in Supplementary Table 3. Rb, retinoblastoma protein; SEM, standard error of the mean.

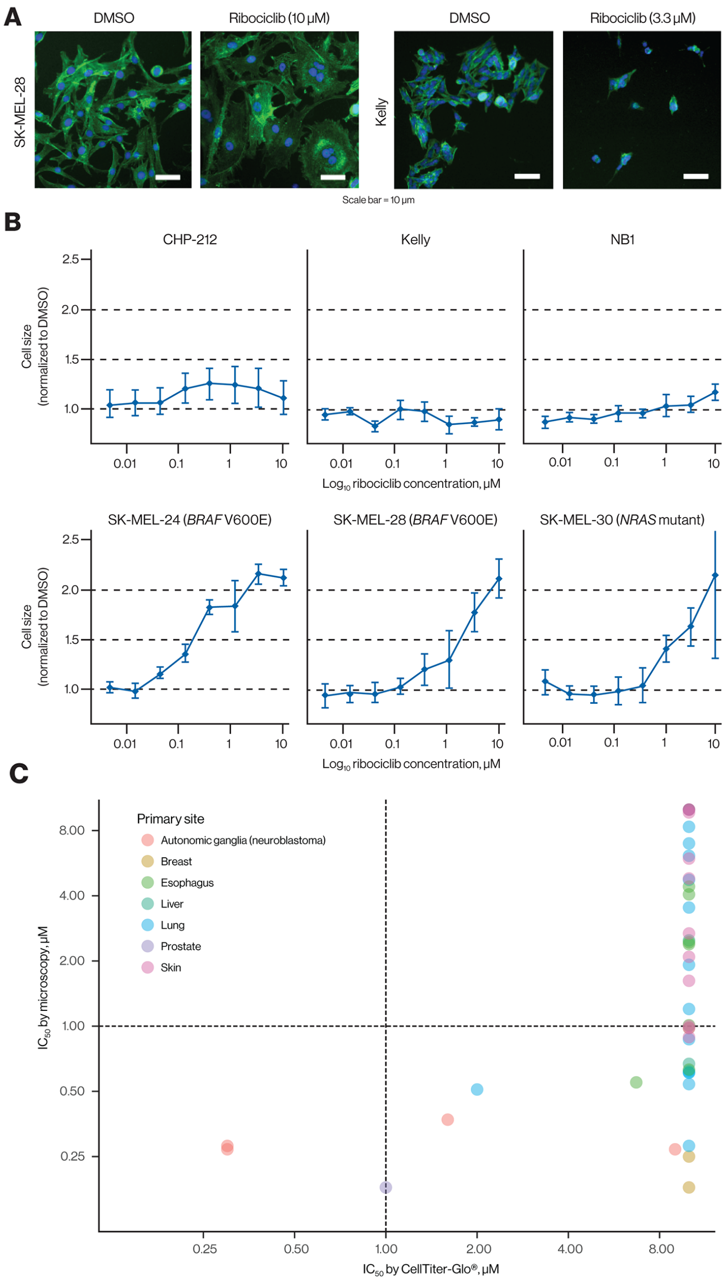

Supplementary Figure 4: Importance of assay principle for assessing antiproliferative effects of ribociclib *in vitro*. (A) As described in Figure 2A, cells after 72-hour ribociclib treatment (3.3 or 10 µM) vs dimethyl sulfoxide (DMSO; original magnification × 10; tubulin [green] and DNA/nuclei [blue] stained with anti–-α-tubulin FITC antibody and Hoechst 33342, respectively). **(B)** Relative cell size change with ribociclib treatment for 3 days in relation to DMSO controls. Error bars show the standard error of the mean. **(C)** Comparative representation of IC_{50} using values for inhibition of proliferation in response to ribociclib, determined by either microscopy or CellTiter-Glo®, in 49 cell lines. Data are also shown in Supplementary Table 4. FITC, fluorescein isothiocyanate; IC_{50}, half-maximal inhibitory concentration.

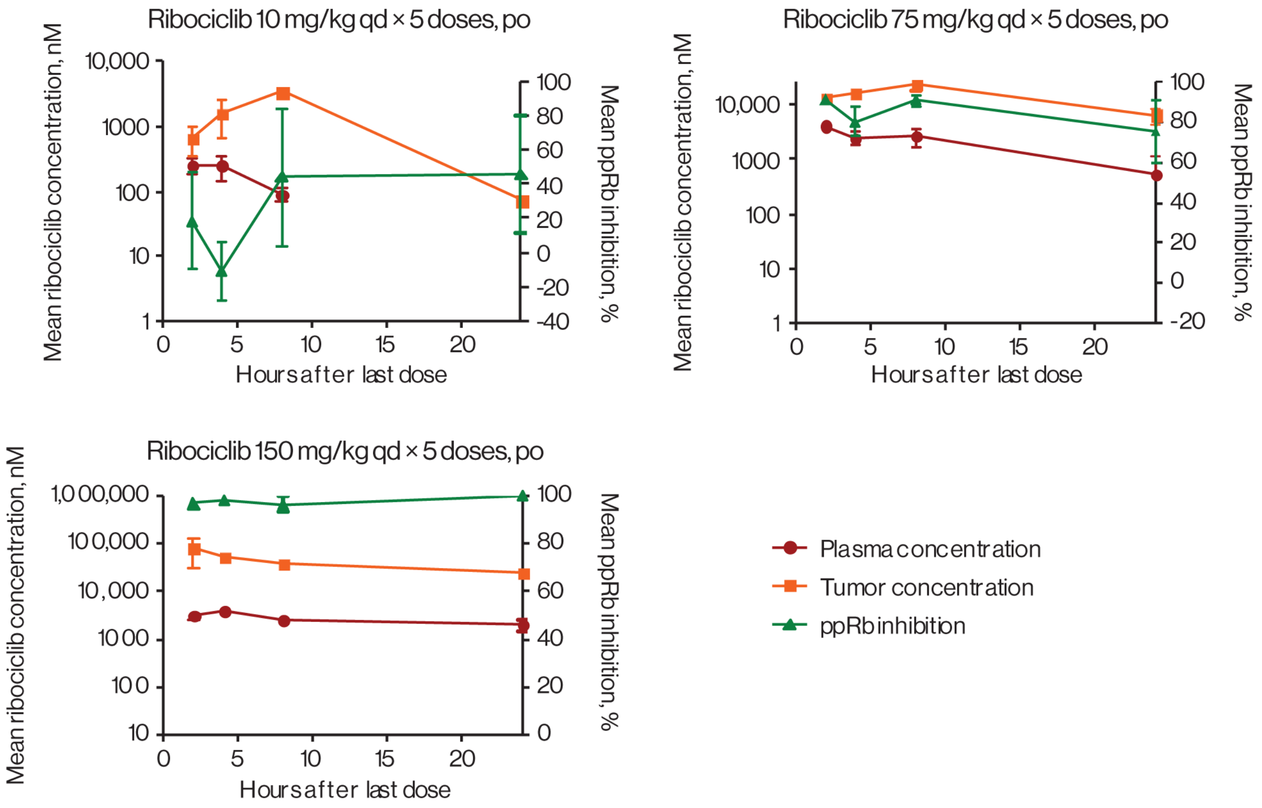

Supplementary Figure 5: Correlation of pharmacodynamic effects (Rb phosphorylation) and pharmacokinetics of ribociclib in nude rats bearing JeKo-1 tumor xenografts. After 5 doses of oral ribociclib at 10 mg/kg (top left line graph), 75 mg/kg (top right line graph), and 150 mg/kg (bottom line graph) daily, tumors were excised at various time points after the last dose (2, 4, 8, and 24 h; n=3 for each time point) for pharmacodynamic analysis (inhibition of Rb phosphorylation by enzyme-linked immunosorbent assay). In addition, plasma samples were collected at 2, 4, 8, and 24 h for assessment of ribociclib concentration. Error bars show the standard error of the mean. po, orally; qd, once daily; ppRb, phospho-Rb protein (Ser780).

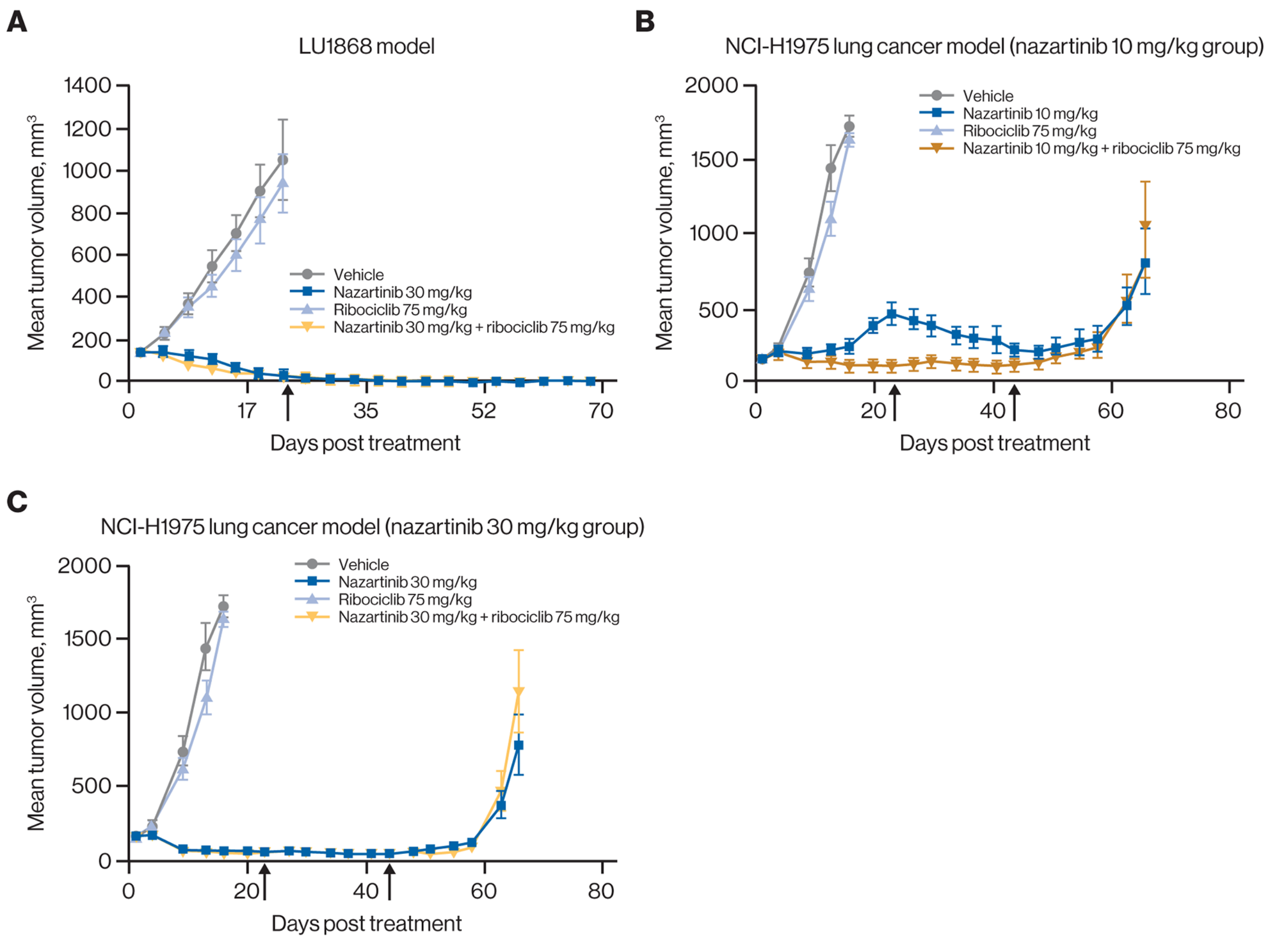

Supplementary Figure 6: Ribociclib *in vivo* combination with nazartinib. **(A)** *In vivo* combination study using the LU1868 patient-derived xenograft model treated with the indicated regimens (n=8 per group). The arrow indicates end of treatment (combination treatment was terminated on day 112). **(B)** *In vivo* combination study using xenografts of the NCI-H1975 cell line treated with the indicated regimens (nazartinib 10-mg group; n=10 per group). The arrows indicate end of treatment. **(C)** *In vivo* combination study using xenografts of the NCI-H1975 cell line treated with the indicated regimens (nazartinib 30-mg group; n=10 per group). The arrows indicate end of treatment. Error bars show the standard error of the mean.

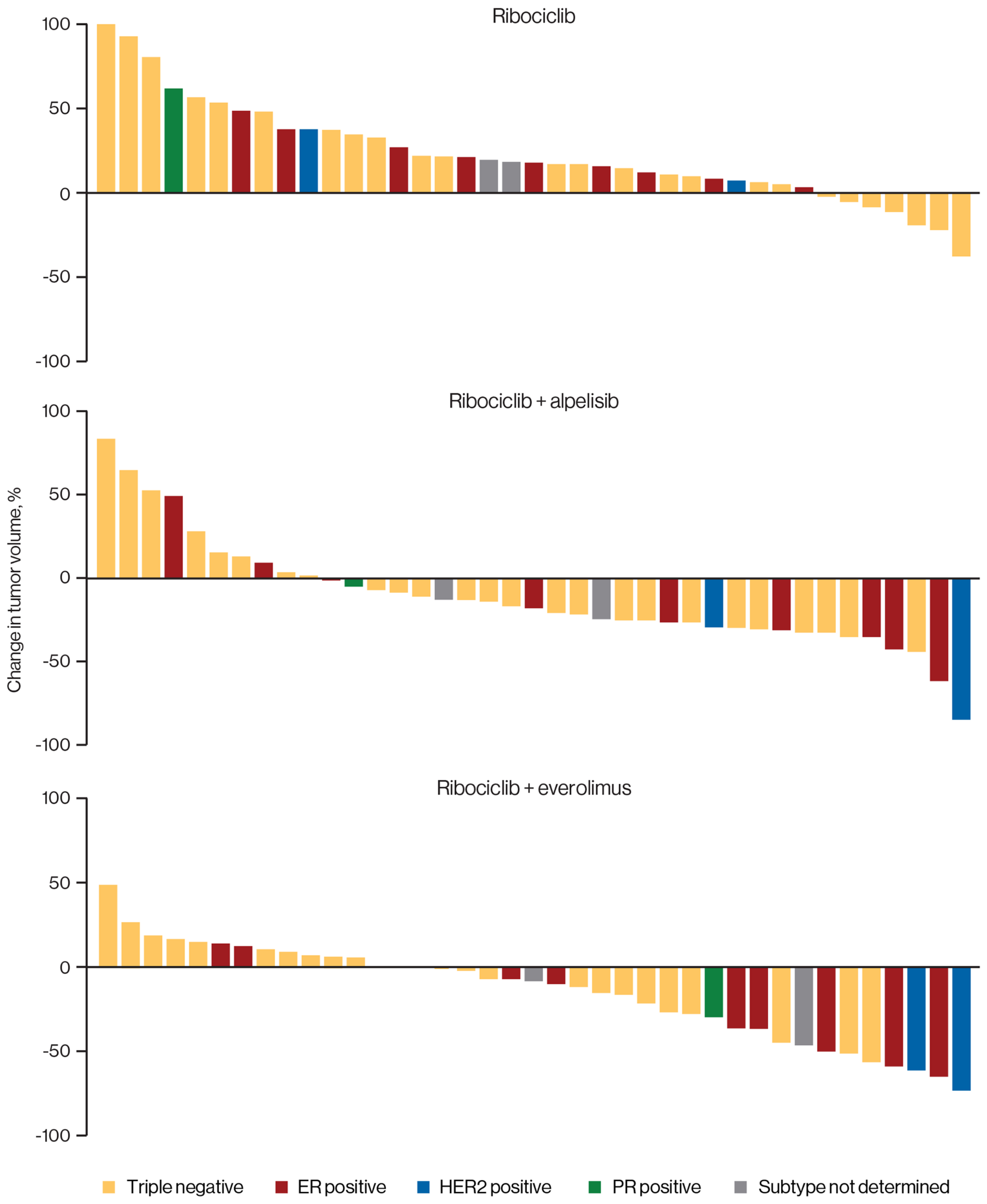

Supplementary Figure 7: Waterfall plots of the response of breast cancer patient–derived xenografts to therapy. Response to ribociclib monotherapy or indicated combinations, as previously described, is shown [7]. Patient-derived xenograft models HBRX1004, HBRX1286, and HBRX1600 were also part of the experiments summarized in Supplementary Table 6. ER, estrogen receptor; HER2, human epidermal growth factor receptor 2; PR, progesterone receptor.

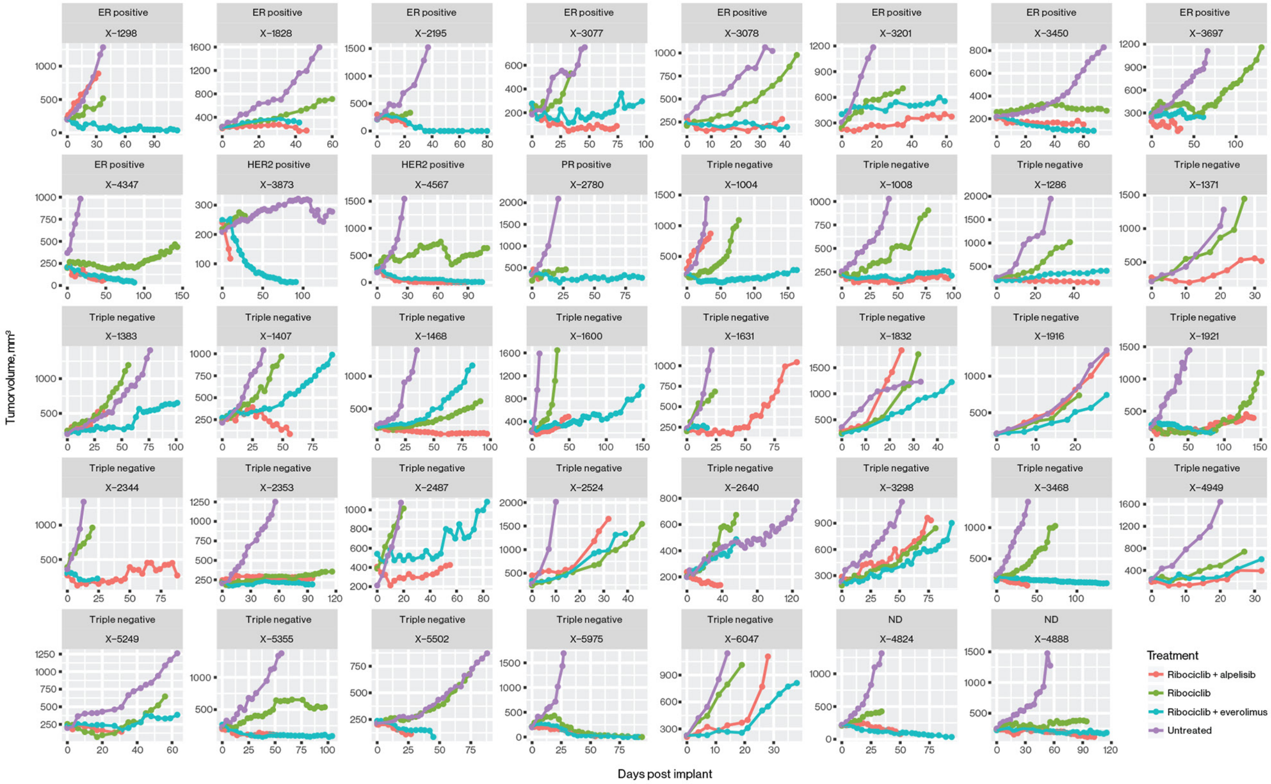

Supplementary Figure 8: Individual tumor growth curves. Tumor volumes over time are shown in each model and treatment condition as included in Supplementary Figure 7, as well as volumes of untreated tumors [7]. ER, estrogen receptor; HER2, human epidermal growth factor receptor 2; ND, subtype not determined; PR, progesterone receptor.

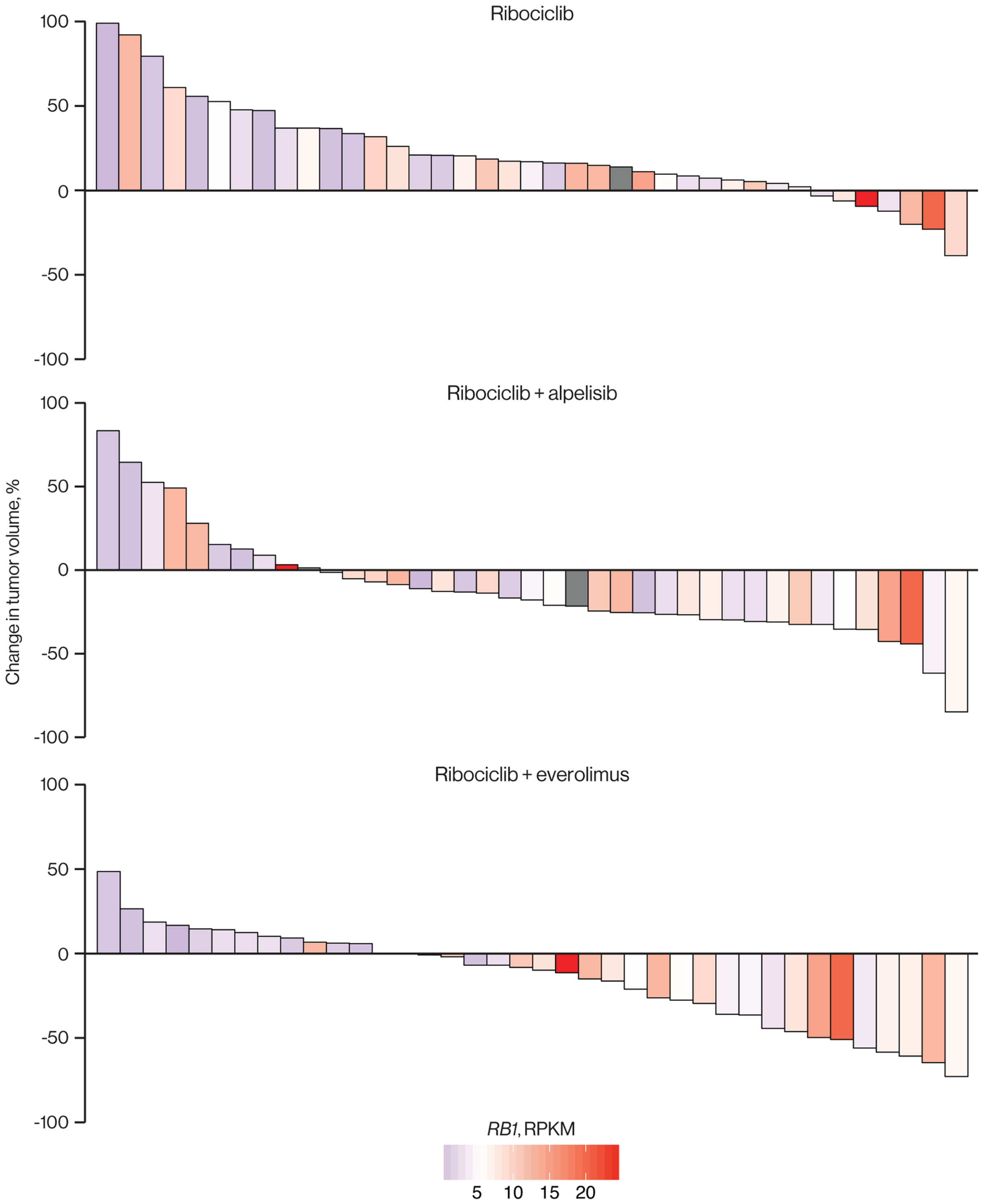

Supplementary Figure 9: Waterfall plots of the response of breast cancer patient–derived xenografts to therapy according to mRNA expression. Waterfall plots as described in Supplementary Figure 7 are shown, but with patient-derived xenograft models colored according to mRNA expression of *RB1*, as determined by RNA sequencing (reads per kilobase million [RPKM]). Red indicates high expression of *RB1*, blue indicates low *RB1* transcript, white indicates the midpoint of the color scale (which reflects the median), and gray indicates that *RB1* expression was not determined.

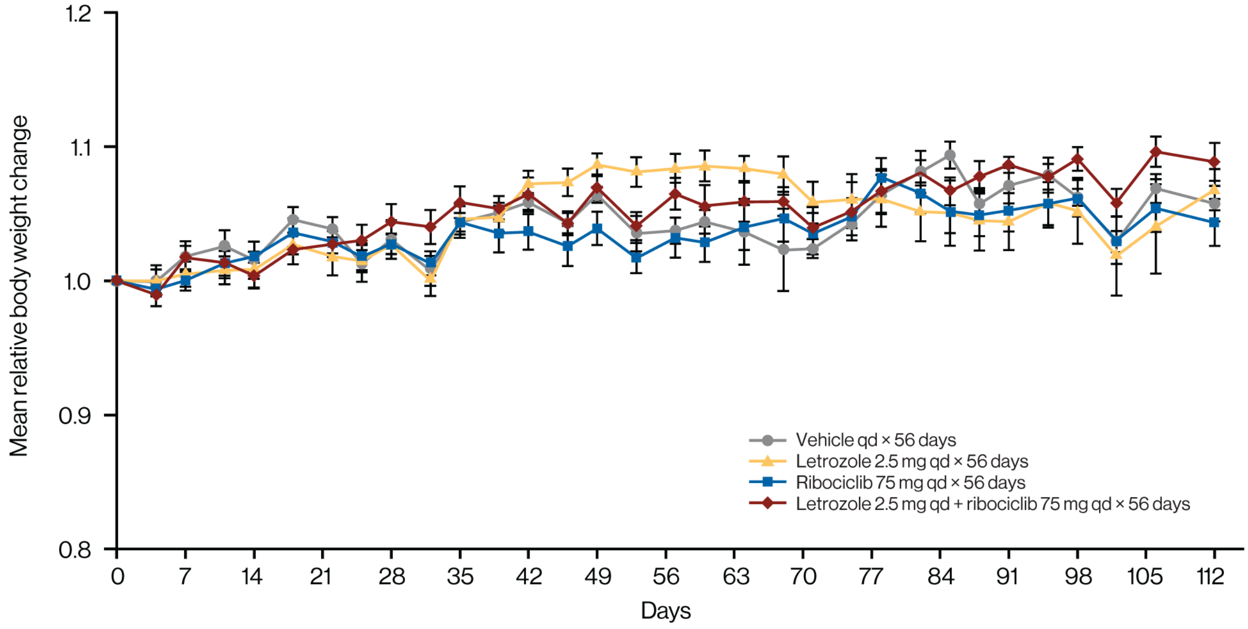

Supplementary Figure 10: Body weight change. Shown is body weight change in the experiment examining antitumor effects of ribociclib in combination with letrozole. Error bars show the standard deviation. qd, once daily.

Supplementary Table 1A: Dissociation constant (Kd) values for the indicated kinases or kinase-cyclin pairs in the KINOME*scan*® assay format

	Ribociclib	Palbociclib	Abemaciclib
**Assay**	**Kd (nM)**	**Kd (nM)**	**Kd (nM)**
CDK4	380	150	41
CDK4-cyclin D1	1.5	1.9	0.078
CDK4-cyclin D3	0.46	1.2	0.076
CDK6	250	33	8.9

Supplementary Table 1B: Raw data of DiscoveRx KINOME*scan* selectivity panel. Numbers represent the percentage of control, where a negative control (dimethyl sulfoxide only) establishes the “100% of control” top signal, and a positive control (promiscuous kinase inhibitors) establishes the “0% of control” baseline. Values were derived from 2 replicates with manual curation for likely technical artifacts.

See Supplementary File 1

Supplementary Table 1C: List of hits (ie, kinases where percentage of control was <35%) for all tested compounds and concentrations. CDK4 assays appear in the shaded area.

See Supplementary File 1

Supplementary Table 2: mRNA expression data and RNA interference dependency profiles for *CDK4*, *CDK6*, *CCND1*, and *CCND3* across 398 cell lines from the Cancer Cell Line Encyclopedia. mRNA expression was measured on Affymetrix™ U133 Plus 2.0 arrays. Data are robust multiarray average normalized and $\log_2$ transformed. RNA interference dependency profiles stem from a large-scale, deep-coverage pooled short hairpin RNA screen [4]. Values represent quantile-normalized log fold changes for 20 individual short hairpin RNAs per gene, aggregated with the ATARiS algorithm [5]. Negative values indicate strong dependencies.

See Supplementary File 1

Supplementary Table 3: Data of cancer cell line profiling using ribociclib or palbociclib. The definition of parameters was previously described in Barretina et al [6]. A_{max}, maximal effect level; EC_{50}, half-maximal effective concentration; IC_{50}, half-maximal inhibitory concentration.

See Supplementary File 1

Supplementary Table 4: IC_{50} values for ribociclib using 2 different methods (CellTiter-Glo® [CTG] and microscopy [MIC]) in 49 cancer cell lines

Cell line	Primary site	Histology	MIC	CTG
KELLY	autonomic_ganglia	neuroblastoma	0.27	0.3
SK-N-SH	autonomic_ganglia	neuroblastoma	0.27	9.0
CHP-212	autonomic_ganglia	neuroblastoma	0.28	0.3
NB1	autonomic_ganglia	neuroblastoma	0.37	1.6
MCF-7	breast	carcinoma	0.18	10.0
T-47d	breast	carcinoma	0.25	10.0
MDA-MB-415	breast	carcinoma	0.98	10.0
BT-483	breast	carcinoma	10.00	10.0
SNU-761	liver	hepatocellular carcinoma	0.67	10.0
SNU-387	liver	hepatocellular carcinoma	2.49	10.0
MGH051	lung	carcinoma	0.28	10.0
NCI-H3122	lung	carcinoma	0.51	2.0
MGH049	lung	carcinoma	0.54	10.0
NCI-H1792	lung	carcinoma	0.61	10.0
NCI-H1573	lung	carcinoma	0.61	10.0
NCI-H460	lung	carcinoma	0.62	10.0
HCC-4006	lung	carcinoma	0.87	10.0
A549	lung	carcinoma	1.20	10.0
COR-L105	lung	carcinoma	1.92	10.0
NCI-H1975	lung	carcinoma	3.53	10.0
NCI-H441	lung	carcinoma	4.74	10.0
HCC-827	lung	carcinoma	6.12	10.0
PC-14	lung	carcinoma	6.98	10.0
NCI-H2030	lung	carcinoma	8.33	10.0
NCI-H2228	lung	carcinoma	10.00	10.0
NCI-H23	lung	carcinoma	10.00	10.0
NCI-H3255	lung	carcinoma	10.00	10.0
KYSE150	esophagus	carcinoma	0.55	6.7
OE33	esophagus	carcinoma	0.63	10.0
TE-10	esophagus	carcinoma	1.01	10.0
TE-5	esophagus	carcinoma	2.39	10.0
TE-8	esophagus	carcinoma	2.44	10.0
KYSE30	esophagus	carcinoma	4.06	10.0
TE-11	esophagus	carcinoma	4.41	10.0
KYSE450	esophagus	carcinoma	10.00	10.0
TE-14	esophagus	carcinoma	10.00	10.0
LnCAP	prostate	carcinoma	0.18	1.0
VCAP	prostate	carcinoma	10.00	10.0
MELJUSO	skin	malignant_melanoma	0.89	10.0
SK-MEL-28	skin	malignant_melanoma	0.98	10.0
SK-MEL-30	skin	malignant_melanoma	1.00	10.0
hs944T	skin	malignant_melanoma	1.62	10.0
SK-MEL-5	skin	malignant_melanoma	2.09	10.0
A-375	skin	malignant_melanoma	2.68	10.0
SK-MEL-2	skin	malignant_melanoma	4.81	10.0
ipc-298	skin	malignant_melanoma	5.94	10.0
UACC-62	skin	malignant_melanoma	9.70	10.0
A2058	skin	malignant_melanoma	10.00	10.0
WM-115	skin	malignant_melanoma	10.00	10.0

Values of 10 indicate that no IC_{50} could be determined in the tested concentration range up to 10 μM.

Supplementary Table 5: Pharmacokinetic and pharmacodynamic parameters from a study of ribociclib administered in nude rats harboring JeKo-1 xenografts

Species	Daily dose, mg/kg	C_{max}, µg/mL	Plasma AUC, mg × h/mL	ppRb inhibition, range
Rat	10	0.11	0.94	0%–46% (24 h)
Rat	75	1.74	20.54	75%–90% (24 h)
Rat	150	1.68	27.02	97%–100% (24 h)

Data obtained after daily doses of ribociclib (10, 75, and 150 mg/kg) for 5 days. Tumors were excised (for PK/PD analyses) and plasma samples were collected (for assessment of plasma concentration of ribociclib) at 2, 4, 8, 24, and 48 h (n = 3 for each time point). AUC, area under the concentration-time curve; C_{max}, maximum plasma concentration; PD, pharmacodynamics; PK, pharmacokinetics; ppRb, phospho-Rb protein (Ser780).

Supplementary Table 6: Summary of mouse xenograft studies with ribociclib as a single agent

Xenograft	Cancer type	Key model features	Dose	T/C
HBRX1286	Breast	TNBC, $PIK3CA^{N345K}$, Rb absent	250 mg/kg qd	82
HBCx-14	Breast	TNBC, Rb absent	125 mg/kg bid × 21	92
HBCx-1	Breast	$HER2^{High}$, Rb absent	125 mg/kg bid × 21	80
HBCx-2	Breast	TNBC, Rb absent	125 mg/kg bid × 21	102
HBCx-3	Breast	TNBC, Rb present	125 mg/kg bid × 21	45
HBCx-10	Breast	TNBC, Rb absent	125 mg/kg bid × 21	82
HBCx-15	Breast	TNBC, Rb absent	125 mg/kg bid × 21	108
HBRX1004	Breast	TNBC, Rb present	250 mg/kg qd	10
HBRX1600	Breast	TNBC, $CDKN2A^{DEL}$, Rb present	250 mg/kg qd	28
HBCx-16	Breast	$HER2^{High}$, Rb present	125 mg/kg bid × 21	71
HBCx-6	Breast	TNBC, Rb present	125 mg/kg bid × 21	64
HBCx-12	Breast	TNBC, Rb present	125 mg/kg bid × 21	33
HBCx-8	Breast	TNBC, Rb present	125 mg/kg bid × 21	10
HBCx-11	Breast	TNBC, Rb present	125 mg/kg bid × 21	52
HCOX1290	Colorectal	$CDKN2A^{DEL}$, $KRAS^{G13D}$, Rb present	250 mg/kg qd	22
HMEX1906	Melanoma	$BRAF^{V600E}$, $CDKN2A^{DEL}$, Rb present	250 mg/kg qd	-12
Hs 944.T	Melanoma	$NRAS^{Q61K}$, $CDKN2A^{H83Y}$	150 mg/kg qd	36
	Melanoma	$NRAS^{Q61K}$, $CDKN2A^{H83Y}$	250 mg/kg qd	19
MEL-JUSO	Melanoma	$NRAS^{Q61L}$, $CDKN2A^{DEL}$	75 mg/kg qd	47
	Melanoma	$NRAS^{Q61L}$, $CDKN2A^{DEL}$	150 mg/kg qd	32
	Melanoma	$NRAS^{Q61L}$, $CDKN2A^{DEL}$	250 mg/kg qd	2
HPAX0991	Pancreatic	$CDKN2A^{DEL}$, $KRAS^{G12D}$, Rb present	250 mg/kg qd	11
HPAX1199	Pancreatic	$KRAS^{G12V}$, Rb present	250 mg/kg qd	18
HPAX1317	Pancreatic	$KRAS^{G12V}$, Rb present	250 mg/kg qd	32
HPAX1633	Pancreatic	$CDKN2A^{DEL}$, $KRAS^{G12D}$, Rb present	250 mg/kg qd	30

Key model features include breast cancer subtype, selected genetic alterations, and retinoblastoma protein (Rb) status as assessed by immunohistochemistry, where available. T/C, treatment/control.
